# Performance optimization of rigid pavement concrete using metakaolin treated RCA and silica fume with an experimental and machine learning based approach

**DOI:** 10.1038/s41598-025-31082-2

**Published:** 2025-12-24

**Authors:** Tariq Alqubaysi, Inamullah Inam, Muhammad Zeeshan Qureshi, Tariq Ali, Khaled Mohamed Elhadi, Imran Haider, Muwaffaq Alqurashi, Mohammad Essa Tanha

**Affiliations:** 1 Department of Civil Engineering, College of Engineering, Northern Border University, 73222 Arar, Saudi Arabia; 2 Department of Civil Engineering, Engineering Faculty, Laghman University, Mehtarlam, Afghanistan; 3 Department of Civil Engineering, University of Engineering and Technology, Taxila, Pakistan; 4 Department of Civil Engineering, Swedish College of Engineering and Technology, Wah, 47080 Pakistan; 5 Civil Engineering Department, College of Engineering, King Khalid University, Abha, Saudi Arabia; 6 Center for Engineering and Technology Innovations, King Khalid University, 61421 Abha, Saudi Arabia; 7 Department of Civil Engineering, College of Engineering, Taif University, P.O. Box 11099, 21944 Taif, Saudi Arabia; 8 Department of Civil Engineering, Engineering Faculty, Nangarhar University, Jalalabad, Afghanistan

**Keywords:** Recycled Concrete Aggregates, Silica fume, Supplementary Cementitious Materials, Machine Learning, Random Forest, K-Nearest Neighbor, Adaptive Boosting, Extreme Gradient Boosting, Engineering, Materials science

## Abstract

Concrete production has a drastic effect on the environment with ordinary portland cement (OPC) contributing approximately 6–8% to the world CO_2_ emissions. The best solutions to reduce these effects is the use of recycled concrete aggregate (RCA) and secondary cementitious materials (SCM) as an alternative to natural aggregates and OPC. Nevertheless, RCA based on low-strength parent concrete is normally characterized by high porosity, low-bond mortar and low mechanical performance which restricts the scope of its structural use. This paper examines the improvement of RCA-based concrete using metakaolin (MK) slurry treatment and addition of silica fume (SF) at different dosages (2.5–10%) with the replacement contents of RCA being 0, 50, 75, and 100%. It has been experimentally found that the addition of MK and SF can significantly enhance mechanical strength and durability and adequately address the intrinsic weaknesses of low-grade RCA. The statistical validation with one-way ANOVA showed that all the *P*-values were less than 0.05 and proved that the improvements due to the addition of SCM and the adjustment of RCA were significant. In addition, 96 experimental and 48 literature-based datasets were used to predict and optimize compressive strength with the use of machine learning (ML) models. K-fold cross-validation was used to fine-tune hyperparameters and the Grey Wolf Optimizer (GWO) was used to optimize them. Extreme Gradient Boosting (XGB) gave the best accuracy with the highest R^2^ of 0.949 (training) and 0.899 (testing) and low RMSE of 1.490 and 1.845 respectively. AdaBoost (ADB) also provided satisfactory results after XGB (R^2^ = 0.929 training, 0.878 testing). In general, the findings substantiate the claim that the ensemble learning frameworks especially XGB are quite effective to derive complex relationships in RCA-based concrete data. RCA, SCMs (MK and SF) and predictive ML modeling can provide a sustainable mix design optimization route and structural life enhancement of RCA concrete in rigid pavement applications.

## Introduction

Concrete is the most widely used construction material in the world and is critical to infrastructure development with an annual production amounting to about 20 billion metric tons, or about 1.6 m^3^ of concrete per capita^1–4^. The production of concrete involves the making of Portland cement as a binder, the production of which causes a substantial negative impact on the environment, consuming 6 to 8% of CO₂ emissions globally^4,5^. Cement-related emissions have been increasing continuously, with an average annual growth rate of 1.5% from 2015 to 2021^6^. Forecasts suggest that the situation is only going to get worse, with as much as 6000 mt/y being needed by 2050^7^. This intensification raises real issues about sustainability and the requirement of eco-efficient solutions for the cement world. To face these environmental and resource constraints, the construction industry is progressively turning to the use of supplementary cementitious materials (SCMs) and alternative aggregates to minimize its use of cement and adopt the principles of the circular economy. Use of industrial waste materials such as fly ash (FA), silica fume (SF), rice husk ash (RHA) and more importantly, recycled concrete aggregate (RCA) has been recognized as a promising solution to promote the sustainable of concrete^8–12^. RCA, a product of demolition waste, has been effectively utilized as a substitute for natural coarse aggregates in structural and pavement quality concretes.

This approach not only alleviates the reliance on new aggregates, but it also helps to reduce the volume of construction and demolition waste; thus, two environmental problems are resolved at once. Recent trends in concrete pavement engineering have turned the focus to the use of waste materials available locally to prepare durable and mechanically stable rigid pavements^9,13,14^. RCA is one such alternative that has potential to minimize the environmental impact of rigid pavement without compromising structural adequacy. However, despite the worry of inferior interfacial transition zones (ITZs) and relatively high porosity of RCA, it has been proved that those shortcomings could be compensated by blending RCA with performance-improving additives, for instance, pozzolanic materials and nanoparticles^15–19^. The addition of SCMs including FA, SF, MK, and RHA had favorable effect for improving the performance and reducing the environmental burden in concrete pavement. These materials enhance workability, long-term strength development and durability by being involved in pozzolanic reactions that refine pore structure and decrease permeability^11,12,19–22^. In the use of concrete pavements where durability and long life are of importance and the effects of environmental detrimental conditions are to be minimized, in general, SCMs for an improved resistance to chloride penetration, sulfate attack and frost are available. For example, SF enhances the density of ITZ and hence, improves the compressive and flexural strengths, fly ash will increase workability, strength at long ages and. While fly ash can retard early strength development, its combination with a faster reacting SCM, such as SF or nano-silica, can influence the setting time and mechanical properties. These advantages make SCMs particularly noteworthy in the design of rigid pavement, in which structural performance and durability must be balanced^16,19,23^.

The adoption of environmentally conscious practices for mitigating the ecological impacts of concrete production drives the need for innovative solutions, especially those that facilitate the sustainable use of RCA from low-strength parent concrete. Earlier works by Poon et al.^24^ report improvements on RCA attributes by means of mechanical, chemical, or mineral admixture treatments; however, the persistent optimization of real-world performance parameters still poses a challenge. To address this challenge, the present study combines experimental bench work with data-driven modeling grounded in machine learning (ML) methodologies to capture and analyze the intricate, nonlinear interactions among the constituents of concrete mixes, treatment parameters, and associated performance metrics^25,26^. With this dataset, ML can swiftly evaluate and estimate the compressive strength and durability across various replacement levels of RCA with SF as well as within a more extensive dataset comprising 48 literature-derived samples. This accelerates and streamlines resource-efficient sustainable decision processes in pavement design^26–28^. The current study adds to the growing discourse on the implementation of digital technologies in concrete engineering while advancing high-performance cement pavements utilizing treated RCA.

### Novelty

The paper presents a synergist innovation in rigid pavement concrete that aims to provide sustainable material development with superior predictive analytics. The majority of previous investigations investigated the surface treatment of recycled concrete aggregate (RCA) or the SCM incorporation separately, unlike this study, which examines the effect of both parameters in one rigid-pavement situation. The past ML-based research on RCA tended to use untreated or uncharacterized aggregates and, therefore, did not indicate the effect of treated RCA structure on mechanical and durability performance. In order to fill this void, metakaolin slurry was added to low-strength (25 MPa) RCA with silica fume (2.5–10%), which enhanced interfacial transition zone, decreased porosity, and strengthened microstructure. The combination of the two strategies made it possible to replace the whole RCA and preserve the pavement-grade performance and durability. More so, the study combines machine learning algorithms, including XGBoost, Random Forest, KNN, and K-Fold with SHAP explainability, to predict and explain the trends in performance based on the experimental data. This research therefore provides both mechanistic and predictive understanding and uplift sustainable pavement engineering to resource efficiency, waste valorization and make use of data to optimize mix.

## Materials and mix design

Ordinary Portland Cement (OPC) ASTM Type-I is used in this study as a primary binder in addition with Metakaolin (MK) and Silica fume (SF) as a secondary cementitious material (SCMs) to overcome the negative effects of recycled concrete aggregate (RCA) as well as MK being utilized in treatment process of RCA. The physio-chemical properties of SF, cement and MK are presented in Table 1.

**Table 1:** Physio-chemical properties of binders.

Parameters	Materials		
	Silica-fume	Cement	Metakaolin
Chemical characteristics			
MgO (%)	0.82	0.9	0.18
Na_2_O (%)	–	–	0.36
TiO_2_ (%)	–	–	1.59
SO_3_ (%)	0.44	2.9	–
Al_2_O_3_ (%)	36.7	6.48	43.1
CaO (%)	0.81	62.94	0.14
Fe_2_O_3_ (%)	1.83	3.68	0.48
SiO_2_ (%)	56.48	21.6	53.2
K_2_O (%)	0.91	–	0.15
Physical characteristics			
Specific surface (cm^2^/g)	–	3542	12,390
Loss on ignition (%)	1.97	1.46	0.73
Specific gravity	2.19	3.07	2.74

Quarried sand with specific gravity of 2.68 as fine aggregates. Both NCA and RCA has the maximum size of 12.5 mm and the specific gravity of 2.73 and 2.24 respectively and a detailed breakdown of sand, NCA and RCA is given in Table 2. The RCA was acquired by breaking down of old concrete having the minimum CS of 25 MPa and then sieved to meet a uniform gradation as NCA. A third generation polycarboxylic superplasticizer similar to Type F of ASTM C-494 was used to maintain the workability of the mixtures.

**Table 2:** Properties of Sand, NCA and RCA.

Parameters	Materials		
	NCA	RCA	Sand
Bulk density (kg/m3)	1664	1419	1591
Water absorption (%)	3.54	4.63	3.86
Abrasion resistance (%)	17.59	29.38	–
Specific gravity	2.81	2.29	2.49
Fineness modulus	3.29	3.46	2.18

The mix proportions is broadly divided into two sections for untreated and treated RCA. A total of sixteen mixtures were prepared in this program and for both section MK was kept constant as 15% while SF varied at 2.5 to 7.5% with an increment of 2.5% as a partial cement replacement and NCA was replaced with RCA as 50, 75 and 100%. For the treated section, RCA was coated with the slurry of MK to enhance the bond between old and new concrete matrix. The choice of experimental parameters was caused by trends which exist in research on sustainable concrete. The concentration of metakaolin was 15% since it was determined to be the most efficient by previous researchers^29–31^ in reducing water absorption and enhancing the density of mixtures containing ITZ in RCA. The introduction of silica fume (2.5–10%) as the variable percentages was done in accordance with works like^32–34^, which showed the largest increase in strengths and durability at less than 10% replacement. RCA replacement levels were chosen to test conservative (0–100%) replacement scenarios which are in line with the recent improvements^35,36^ of giving treated RCA the chance to perform at the same or better performance as natural aggregates with proper conditions. This paper is hence concerned with the effect of SF content at a fixed MK-treated RCA condition and the effects of binder are isolated of the treatment of aggregates.

A detailed mix design of the study is mentioned in Table 3 below:

**Table 3:** Mix design of concrete.

Mix ID	Cement (Kg/m^3^)	MK (Kg/m^3^)	SF (Kg/m^3^)	RCA (Kg/m^3^)	NCA (Kg/m^3^)	Sand (Kg/m^3^)	Water (Kg/m^3^)	SP (Kg/m^3^)
R0-SF2.5	440	6	11	0	1100	600	220	2
R50-SF2.5	440	6	11	550	550	600	220	4
R75-SF2.5	440	6	11	800	300	600	220	6
R100-SF2.5	440	6	11	1000	0	600	220	8
R0-SF5	440	6	22	0	1100	600	220	2
R50-SF5	440	6	22	550	550	600	220	4
R75-SF5	440	6	22	800	300	600	220	6
R100-SF5	440	6	22	1000	0	600	220	8
R0-SF7.5	440	6	33	0	1100	600	220	2
R50-SF7.5	440	6	33	550	550	600	220	4
R75-SF7.5	440	6	33	800	300	600	220	6
R100-SF7.5	440	6	33	1000	0	600	220	8
R0-SF10	440	6	44	0	1100	600	220	2
R50-SF10	440	6	44	550	550	600	220	4
R75-SF10	440	6	44	800	300	600	220	6
R100-SF10	440	6	44	1000	0	600	220	8

## Experimental setup

The experimental program in this study is divided into two portions, laboratory work and machine learning (ML) for the prediction of compressive strength on the basis of laboratory results and data extracted from past literature. Laboratory work is further divided into two groups, untreated recycled concrete aggregate (URCA) and treated RCA. The RCA was treated with metakaolin slurry having water to binder ratio of 1:5 while consisting of 50% cement and 50% metakaolin (MK). The process began of pouring 25 kg of RCA in MK slurry for 30 min followed by vibration after each 10 min cycle. After that, treated RCA was kept at room temperature for a period of 2 days to air dry the RCA before testing. A total of 384 samples were cast, in which 144 samples were used for compressive strength as per ASTM C-39 and 48 samples were used for abrasion test as per ASTM C-944. The concrete was prepared by using a semi-automatic mixing machine having an average rpm of 80 and was mixed for 100 s and then 150 mm × 300 mm cylinders was cast for both compression and abrasion test. After 24 h the mold was then demolded and transferred to the curing tank for the desired period of curing. The compression test used to assess the compressive strength of concrete was performed using the compression machine. For each mix three samples were tested and then average compressive strength was calculated and the results were then statistically evaluated for accuracy. The abrasion test simulates the abrasive conditions applied on concrete which include regular abrasion as it is subjected to such processes as high volumes of traffic on the highways and concrete bridges. Three cylinders for each mix were subjected to a drilling press that used a rotating-cutter. The specimens were initially weighed to their nearest 0.1 g after which it was clamped securely in the rotating-cutter drill press. The constant load of 98 N was applied to the surface of each sample in a 20, 40 60- and 80-min abrasion process. The samples were cleaned after the test using the closed air and re-weighed to the nearest 0.1 g. Mean loss of mass was calculated by weighing the sample (mass) weights at the end minus the mass weights at the beginning.

For the ML section, a total of four algorithms were applied to dataset namely K-Nearest Neighbor (KNN), Random Forest (RF), Adaptive Boosting (ADB) and Extreme Gradient Boosting (XGB). KNN is an instance-based, simple learning algorithm that can be employed as a classifier and regression. It operates by searching for the majority course (in classifying) or the average (in regressing) of the K closest information objects to a particular query object. It lacks a clear training step, thus being computationally cheap when it comes to small datasets. The equation of KNN is given below in Eq. 1:

1 $$\hat{y} = mode \left( {y_{1} ,y_{2} ,y_{3} , \ldots \ldots ,y_{k} } \right)$$

where ${y}_{1}$, ${y}_{2}$, ${y}_{3}$,…, ${y}_{k}$ are the labels of the K nearest neighbors and $\widehat{y}$ is the predicted label.

Random Forest is a method of ensemble learning which builds a set of decision trees during training. It exploits the technique of bagging (bootstrap aggregation) to train several decision trees and average their predictions in order to increase accuracy and minimize overfitting. It can be used especially when working with large datasets and a big number of features. The equation of RF is given below Eq. 2:

2 $$\hat{y} = \frac{1}{N} \mathop \sum \limits_{i = 1}^{N} T_{i} \left( x \right)$$

where ${T}_{i} (x)$ represents the prediction of the i-th decision tree, and $N$ is the number of trees in the forest.

Adaptive Boosting (ADB) is an ensemble algorithm in which a series of weak learners (more often decision trees) strive to produce a strong classifier. The model alters the weights of the mistakenly classified individuals so as to concentrate on hard cases, slowly enhancing the performance of the classifier. The equation of ADB is given below Eq. 3:

3 $$\hat{y} = sign \mathop \sum \limits_{t = 1}^{T} a_{t} h_{t} \left( x \right)$$

where ${a}_{t}$ is the weight of the t-th weak classifier, ${h}_{t }(x)$ is the prediction of the weak classifier, and *T* is the total number of iterations.

Extreme Gradient Boosting (XGB) is a fast as well as efficient gradient booster algorithm and it utilizes the decision tree as a basic learner. It maximizes the model with the first and second-order derivation and uses regularization so as not to overfit the model. XGBoost is known for its speed and performance in large-scale ML tasks. The equation of ADB is given below Eq. 4:

4 $$\lambda \left( {\uptheta } \right) = \mathop \sum \limits_{n = 1}^{n} \ell (y_{i} ,\hat{y}_{i} ) + \mathop \sum \limits_{k = 1}^{K} \Omega (f_{k} )$$

where *ℓ* is the loss function, ${\widehat{y}}_{i}$ is the predicted value, ${f}_{k}$ represents the k-th tree, and Ω ${(f}_{k})$ is the regularization term to prevent overfitting.

## Result and discussion

### Experimental work

#### Compressive strength

**Fig. 1:**
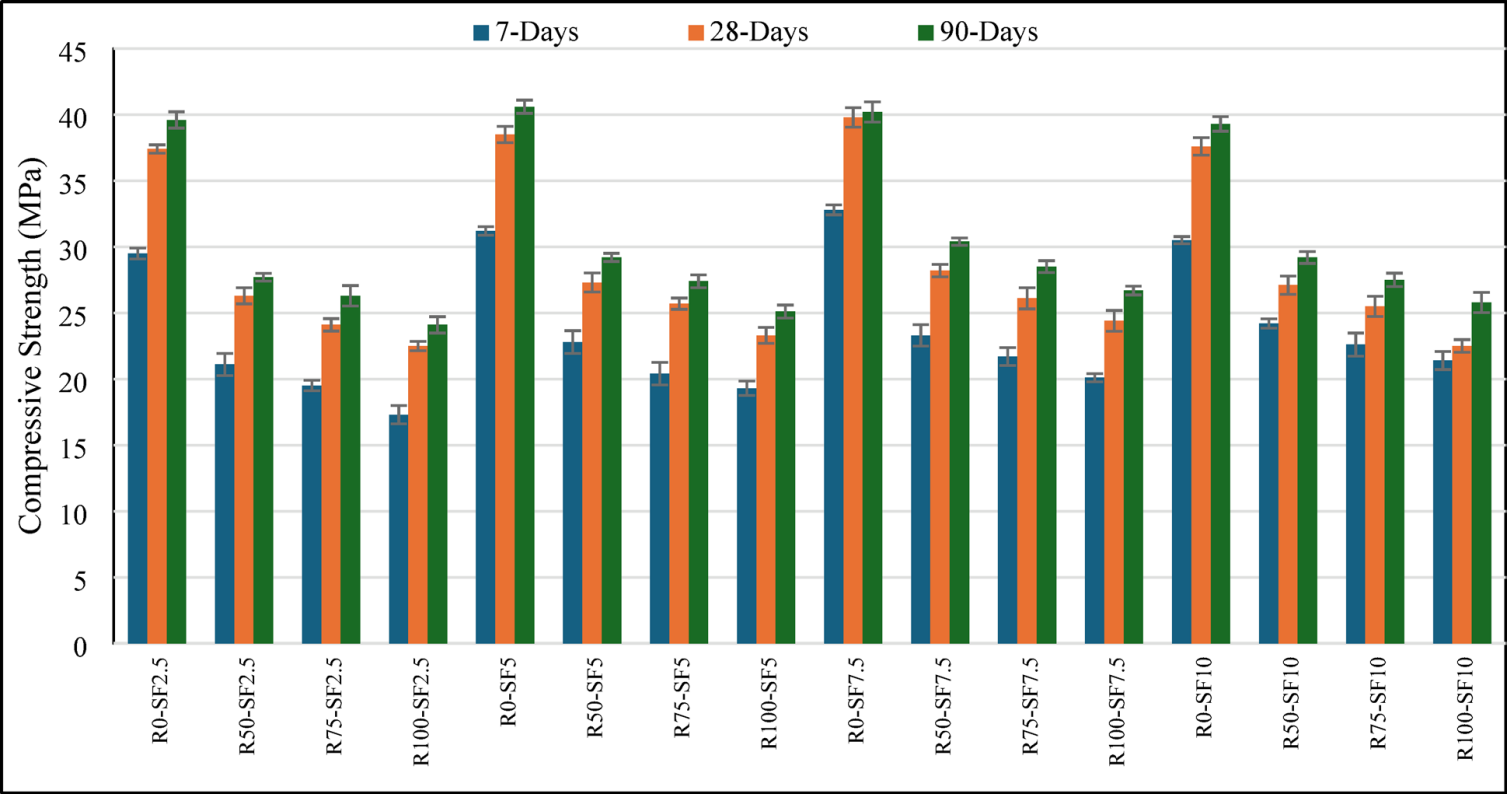
Compressive strength variation of untreated RCA sample at different curing ages.

The compressive strength of concrete is one of the most important features which influences its structural integrity and is particularly crucial for rigid pavements subjected to continuous traffic loads and environmental conditions. This study investigates both untreated recycled concrete aggregate (RCA) and metakaolin slurry treated RCA with 15% constant metakaolin (MK) and varying silica fume (SF) doses of 2.5%, 5%, 7.5%, and 10%, to examine and compare their compressive strength at 7, 28 and 90 days. The untreated RCA series exhibited a significant decrease in compressive strength as RCA content increased as shown in Fig. 1. For 28 days curing, the control blend (R0-SF7.5) yielded a compressive strength of 36.2 MPa, while its counterpart with 100% RCA, R100-SF7.5, reduced to 21.2 MPa. This reduction is consistent with earlier studies that suggest the strength reduction is due to the RCA’s porous structure, weakly bonded adhered mortar, and poor interfacial transition zones (ITZ)^37–39^. Such defects inhibit efficient stress transfer within the matrix, leading to reduced long-term mechanical performance. In addition, the 10% SF blends showed only slight strength improvements (35.2 MPa) relative to controlled sample (34.5) but it is less than 7.5% (36.2 MPa) indicating that optimal dosage thresholds exist, beyond which further enhancement stagnates or even deteriorates, likely due to particle agglomeration or a lack of calcium hydroxide for sufficient pozzolanic reaction^40,41^.

**Fig. 2:**
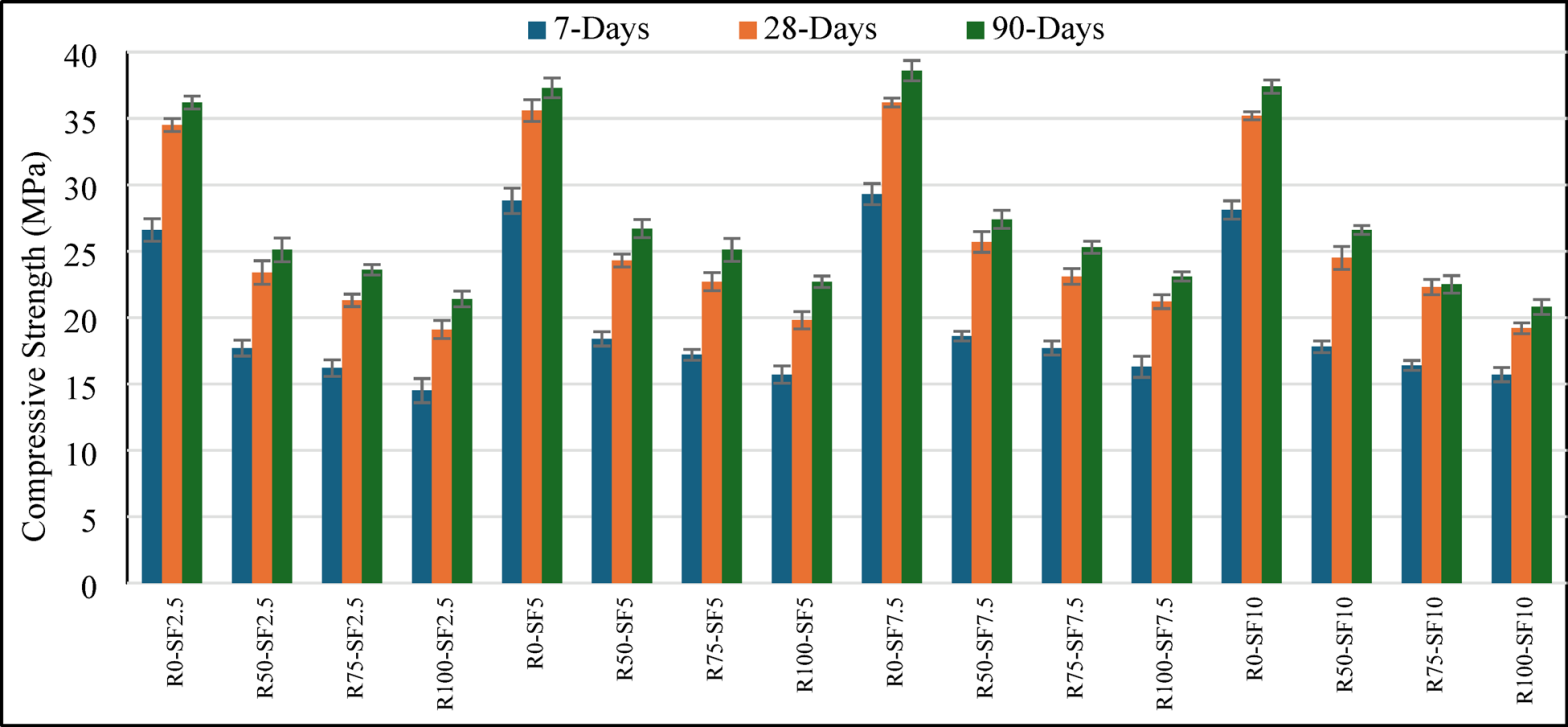
Compressive strength variation of treated RCA sample at different curing ages.

**Fig. 3:**
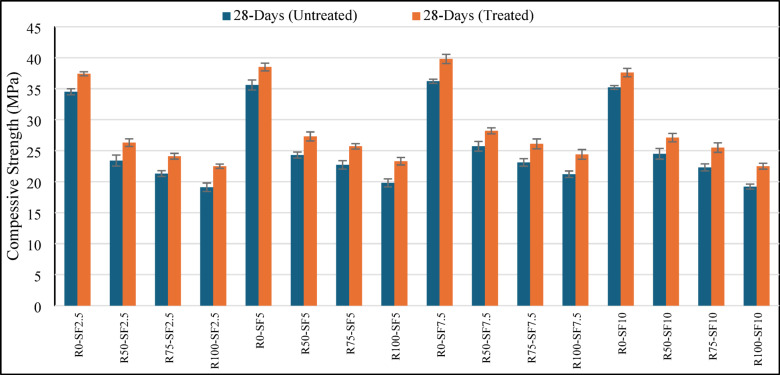
Comparison of 28 days compressive strength of treated & untreated RCA sample.

At all replacement levels, the RCA series treated with MK slurry as represented in Fig. 2 showed notable enhancements. While comparing the 28 days compressive strength the R100-SF7.5 mix improved from 21.2 MPa (untreated) to 24.4 MPa (treated) showing a 13.1% gain which is significant. Also, at both the 50% and 75% RCA substitutions, the strength recovery for the treated mixes was greater, suggesting that the surface MK coating and ITZ refinement strengthened bond failure areas within the microstructure of RCA^42^. MK serves to shield the surface pores of RCA and also participates in secondary pozzolanic reactions to produce additional calcium silicate hydrate (C-S–H) gel which strengthens the bond at the aggregate and paste interface^43,44^. Treatment with MK added synergistically with SF. All levels of RCA resulted in maximum compressive strength with 7.5% SF. As resulted for the R0-SF7.5 blend, it exceeded 39.8 MPa while outperforming both the 5% and 10% SF blends for the curing age of 28 days. Consistent with literature, moderate SF dosages (6–8%) are reported to optimize packing density and pozzolanic activity while not causing issues with workability or dispersion^45,46^. The improvement in microstructure stiffness not only increases early-age strength but also improves long-term performance through reduced permeability and a refined pore structure^47^. From the comparison concrete compressive strength by using the untreated and treated RCA at 28 days of curing as shown in Fig. 3, untreated concrete suffers from severe strength penalties with RCA usage over 50%, while with MK treatment and optimal silica fume content (7.5%), concrete mixes with up to 100% RCA achieved compressive strengths over 26 MPa which meets the requirements for use in rigid pavements. These findings justify the implementation of a combination of approaches using material-level strengthening techniques (treatment with MK) and SCM-based matrix optimization (addition of silica fume) to offset the drawback SF of low-strength RCA. These findings support previous studies showing that pozzolanic mineral supplements can add MK value to overcome the deficiencies of RCA. Thomas et al. pointed out that the use of SCMs with recycled aggregates improves strength and durability indices considerably^48^. Also, Tam et al. reported that the surface treatment of RCA with cementitious slurries improved mechanical performance because the ITZ properties were enhanced^49^. Moreover, more recent studies by Zhang et al. seem to argue that hybrid approaches, especially those employing ML-guided optimization with experimental validation, can more reliably and efficiently predict and improve the performance of RCA-SCM concretes^50^.

#### Abrasion resistance

**Fig. 4:**
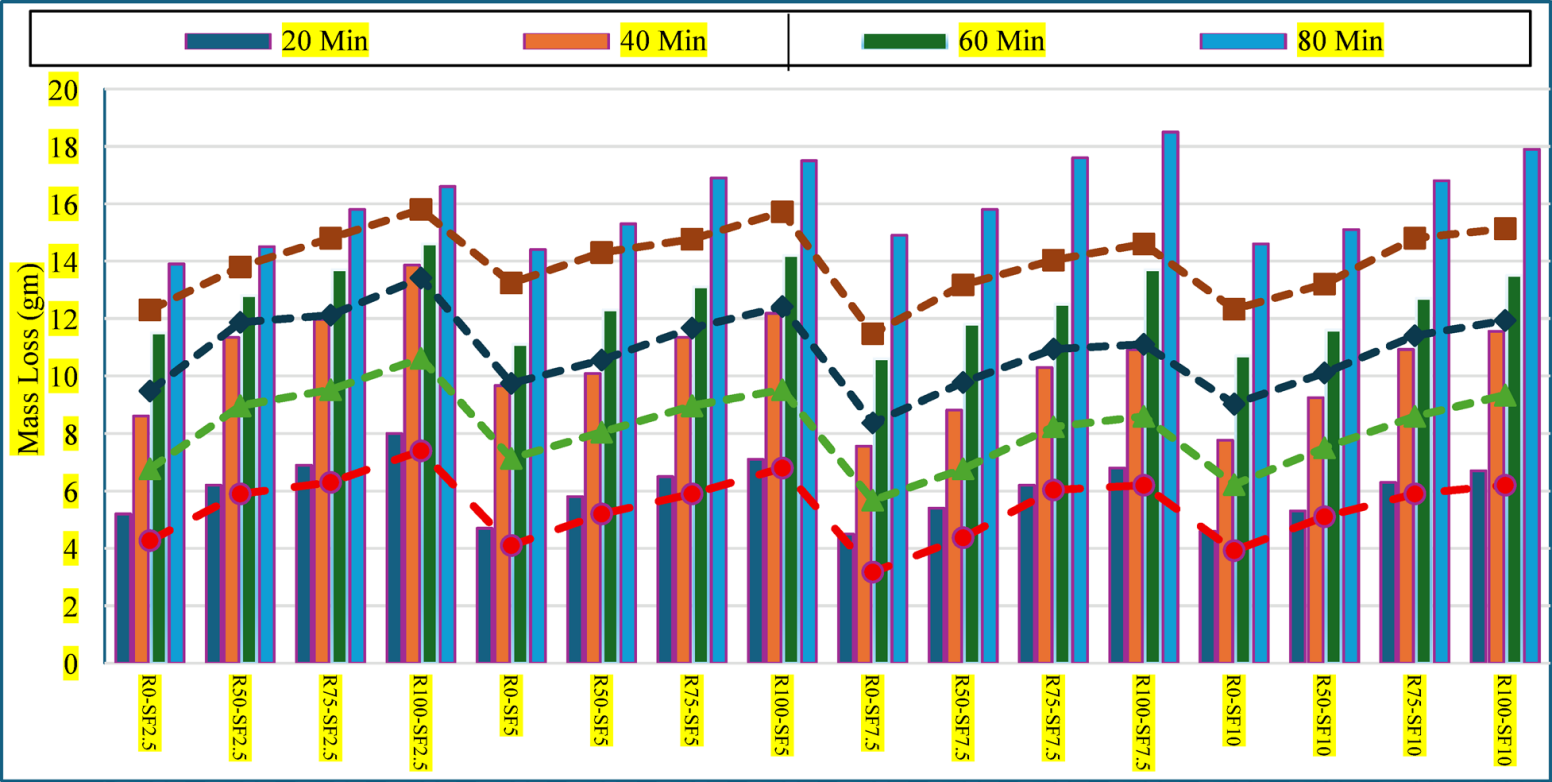
Mass loss of treated & untreated RCA sample over time.

Abrasion resistance is a critical performance indicator for rigid pavement concrete, directly linked to surface durability under vehicular and environmental wear. The current study evaluated the abrasion performance of concrete incorporating varying proportions of untreated and treated recycled concrete aggregate (RCA) and silica fume (SF) for the time interval of 20, 40, 60 and 80 min and the results are plotted in Fig. 4. The findings underscore the significance of synergistic material design strategies for enhancing the abrasion durability of RCA-based pavement concretes. A consistent trend was observed wherein increased RCA content and extended time period led to a progressive decline in abrasion resistance in untreated mixes. For instance, at 2.5% SF, abrasion loss rose from 5m2 gm (R0) to 8 gm (R100) for the period of 20 min and this loss reached 13.9 gm for R0-SF2.5 when time period expands over 80 min, reflecting the porous, microcracked, and weaker nature of untreated RCA. These findings are consistent with prior investigations by Zega and Maio^51^ and Evangelista and Brito^52^ who reported reduced mechanical integrity and wear resistance in concrete containing untreated RCA due to old, adhered mortar and poor interfacial bonding. The incorporation of SF significantly mitigated this degradation hence SF addition enhanced abrasion resistance across all RCA levels, attributed to its pozzolanic activity and micro-filling effect, which refined the pore structure and densified the interfacial transition zone (ITZ). Notably, increasing SF content from 2.5% to 7.5% led to a marked reduction in abrasion loss. For example, in the R100 group, abrasion resistance improved from 8 gm (SF2.5) to 6.8 gm (SF7.5) for the 20 min timespan. Beyond 7.5%, however, the rate of improvement diminished, suggesting a performance plateau likely due to particle agglomeration or reduced workability, consistent with observations by Aydin et al.^53^ and Siddique^54^ on the optimal SF dosage in high-performance concrete.

Metakaolin slurry treatment of RCA further enhanced abrasion resistance, particularly at higher RCA replacements. Treated aggregates contributed to a denser interfacial zone and improved bond with the cement matrix, as evidenced by reductions in abrasion loss across all SF levels. For instance, the R100-SF2.5 mix improved from 8 gm (untreated) to 7.4 gm (treated), while R100-SF7.5 improved from 6.8 gm to 6.2 gm at the cycle of 20 min. These improvements can be attributed to the pozzolanic coating and pore-sealing effect of metakaolin MK, which introduces reactive aluminosilicates that enhance both aggregate integrity and the matrix-aggregate bond^55^. The combined approach of optimal SF inclusion (7.5%) and RCA surface modification yielded the best abrasion performance. The R0-SF7.5 treated mix achieved the lowest abrasion loss (3.2 gm), even surpassing the natural aggregate concrete. This hybrid methodology demonstrates that with proper treatment and mix optimization, RCA-based concrete can not only match but outperform conventional concrete in surface durability metrics. These findings contribute to the evolving paradigm of sustainable concrete design, affirming that durability deficits associated with RCA can be effectively mitigated through SCM synergy and aggregate engineering. Such strategies support the development of eco-efficient rigid pavements with extended service life and reduced life-cycle maintenance.

#### Statistical validation of variance (ANOVA)

The experimental study explored the effect of different levels of replacement of recycled aggregates (RA), Silica fume (SF) content and treated and untreated RCA on the compressive strength and abrasion resistance on the rigid pavement concrete. In order to test the statistical significance of differences between the mechanical and abrasion performance of recycled aggregate concrete (RAC), a one-way Analysis of Variance (ANOVA) was conducted with a 95% confidence interval (α = 0.05). All Concrete mixes were tested and the mean values of concrete mixes together with the standard deviations were used to plot the results with the error bars. The null hypothesis (Ho) was that all mixtures had the same mean performance whereas the alternative (H_1_) was that at least one of them share statistically significant difference. F-values and *P*-values were determined to measure the level of significance of each of the analyzed properties. Tables 4, 5, 6, and 7 are the summaries of the ANOVA results of the compressive strength and abrasion resistance for different SF content and replacement level of treated and untreated RCA results. The F-values were all greater than the critical values of the corresponding parameters with all *P*-values less than 0.05 as a result of this confirming significant performance differences between the 16 mixes. The joint effect of SF content and treatment of RCA generated significant differences in strength as well as abrasion resistance. As such, the combination of 7.5% SF and MK treated RCA was the most desirable ratio of strength and abrasion resistance and thus suitable in terms of sustainable rigid pavement use that requires better performance, especially at 50% RA sustainability. Better suited to non-structural or secondary concrete elements. Besides, the combination of RA and SCMs and treatment promotes sustainable construction methods by reducing the use of natural aggregates and continuing the development of environmentally friendly concrete production.

**Table 4:** ANOVA results for untreated RAC’s CS at different curing ages.

Curing Age	Variation	SS	*df*	*MS*	F	*P*-Value	F-Critical
7 days	Between Groups	1222.689	15	81.51258	190.5177	6.54E-27	1.99199
	Within Groups	13.69113	32	0.427848			
28 days	Between Groups	1714.667	15	114.3111	296.2275	6.12E-30	1.99199
	Within Groups	12.34847	32	0.38589			
90 days	Between Groups	1726.312	15	115.0875	308.8526	3.16E-30	1.99199
	Within Groups	11.92413	32	0.372629

**Table 5:** ANOVA results for treated RAC’s CS at different curing ages.

Curing Age	Variation	SS	*df*	*MS*	F	*P*-Value	F-Critical
7 days	Between Groups	1017.215	15	67.81432	176.6094	2.16E-26	1.99199
	Within Groups	12.28733	32	0.383979			
28 days	Between Groups	1667.501	15	111.1667	294.0206	6.89E-30	1.99199
	Within Groups	12.09893	32	0.378092			
90 days	Between Groups	1543.353	15	102.8902	357.6767	3.08E-31	1.99199
	Within Groups	9.2052	32	0.287663

**Table 6:** ANOVA results for untreated RAC’s AR at different curing time duration.

Time (Min)	Variation	SS	*df*	*MS*	F	*P*-Value	F-Critical
20 min	Between Groups	44.9925	15	2.9995	26.74428	6.75E-14	1.99199
	Within Groups	3.588955	32	0.112155			
40 min	Between Groups	131.7625	15	8.784169	17.45941	2.65E-11	1.99199
	Within Groups	16.09982	32	0.503119			
60 min	Between Groups	68.43	15	4.562	11.2766	8.05E-09	1.99199
	Within Groups	12.94575	32	0.404555			
80 min	Between Groups	90.74813	15	6.049875	5.984045	1.13E-05	1.99199
	Within Groups	32.35203	32	1.011001

**Table 7:** ANOVA results for treated RAC’s AR at different time duration.

Time (Min)	Variation	SS	*df*	*MS*	F	*P*-Value	F-Critical
20 min	Between Groups	61.47508	15	4.098339	45.55813	2.66E-17	1.99199
	Within Groups	2.878671	32	0.089958			
40 min	Between Groups	84.18507	15	5.612338	28.10278	3.31E-14	1.99199
	Within Groups	6.390641	32	0.199708			
60 min	Between Groups	84.90424	15	5.660283	11.00733	1.09E-08	1.99199
	Within Groups	16.45531	32	0.514229			
80 min	Between Groups	71.4252	15	4.76168	6.140605	8.64E-06	1.99199
	Within Groups	24.81413	32	0.775441			

### Machine learning

#### Data analysis and authentication

Commonly for researches, data is acquired either from laboratories experimental results or existing literature to build a model for predictions and statistical analysis. For this research study, a detailed dataset consisting of 144 data points regarding the compressive strength of concrete was gathered by both experimental work and reviewing previous literature, and 96 data points were based on experimental results and the rest was extracted from Elhadi et al., study^56^. The analysis covered nine parameters which were considered as the primary input variables including cement content, metakaolin (MK), fly ash (FA), sand, natural and recycled coarse aggregates, water content, super-plasticizer and curing age which represents the curing period in days. These parameters were essential for predicting the final compressive strength which is the outcome variable. The dataset’s key features are outlined in Table 8, which summarizes a statistical analysis on the mix design of concrete with particular emphasis on its constituent elements and characteristics which include cement, MK, silica-fume (SF), sand, natural coarse aggregate (NCA), recycled concrete aggregate (RCA), water, super-plasticizer, curing age and compressive strength. It explain the most important values such as maximum and minimum values of a variable’s mean, median, standard deviation as well as kurtosis and skewness giving an idea about range with central value of a data set along with its variation shape.

**Table 8:** Statistical analysis of data.

Variables	Maximum	Minimum	Mean	Median	Standard deviation	Kurtosis	Skewness
Cement (Kg/m3)	440	340	423.3	440	40.0	− 22,096	− 1.031
Metakaolin (Kg/m3)	6	0	53.3	6	22.3	0.383	− 1.313
Silica-fume (Kg/m3)	44	0	18.8	16.875	16.8	− 1.371	0.282
Sand (Kg/m3)	600	550	566.7	550	23.7	− 1.511	0.715
Natural coarse aggregate (Kg/m3)	1200	0	500.0	425	419.3	− 1.105	0.436
Recycled concrete aggregate (Kg/m3)	1200	0	616.7	700	402.6	− 1.060	− 0.467
Water (Kg/m3)	225	220	223.3	225	2.4	− 1.511	− 0.715
Super-plasticizer (Kg/m3)	10	0	4.7	5	3.8	− 1.446	− 0.039
Curing age (Days)	90	7	42.4	28	34.7	− 1.503	22,050
Compressive strength (MPa)	41.5	14.5	26.0	25.1	6.4	− 0.318	22,073

**Fig. 5:**
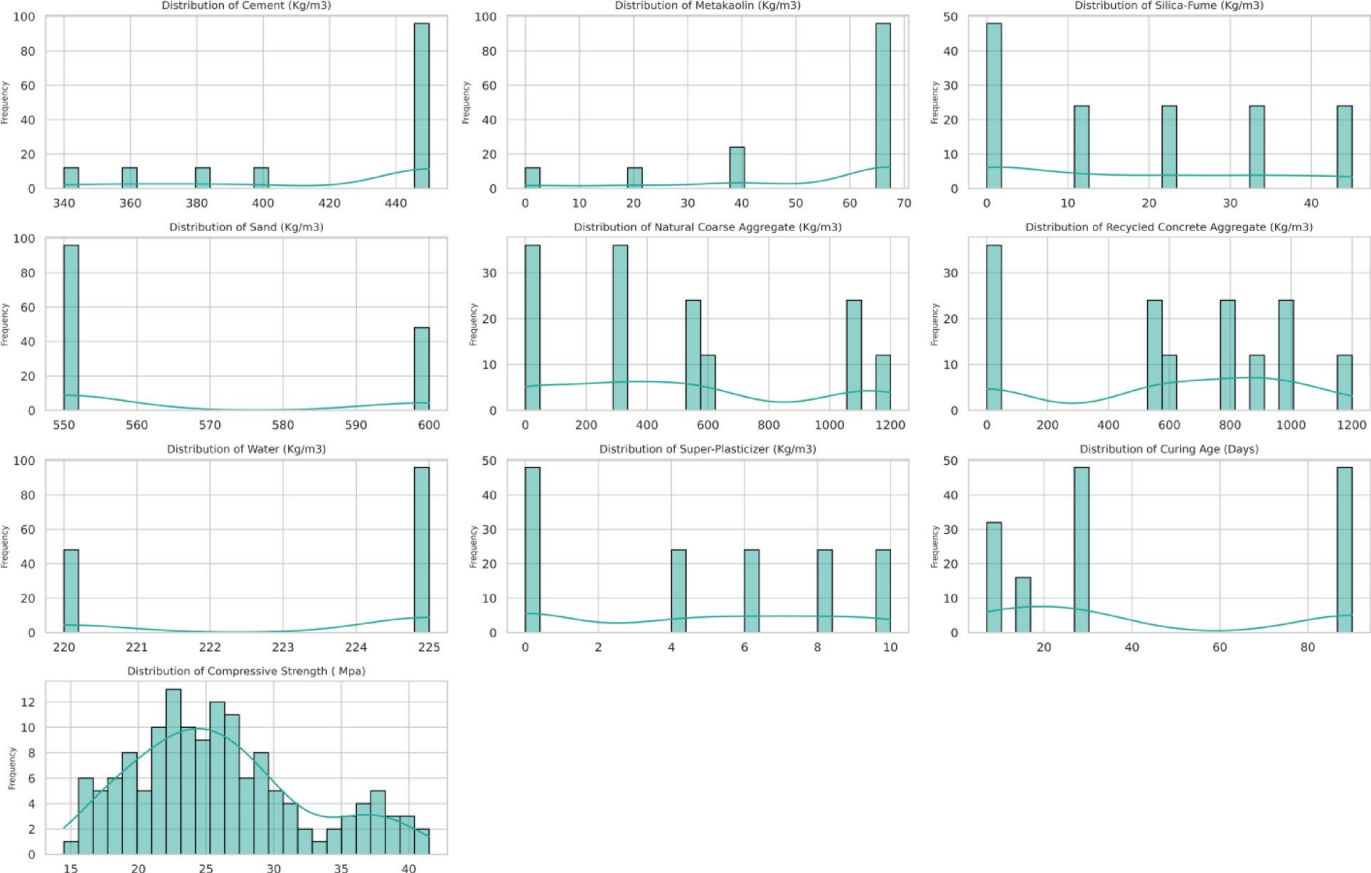
Distribution graph of input and output variables.

Through the distribution plots, as shown in Fig. 5, the shape of each variable’s distribution can be understood along with its normality, skewness and outliers. Some of the variables consisting of MK, Cement, Sand, Water and Super-Plasticizer display strong positive skewness. On the other hand, Natural Coarse Aggregate, Curing Age and RCA show some sort of symmetry in their distributions. Compressive Strength appears to be almost normally distributed with a peak at 25–30 MPa. It seems that outliers are most prominent in features that draw from positively skewed distributions like Cement or Water, which appear to signal scarcity when it comes to higher values within concrete mixes.

**Fig. 6:**
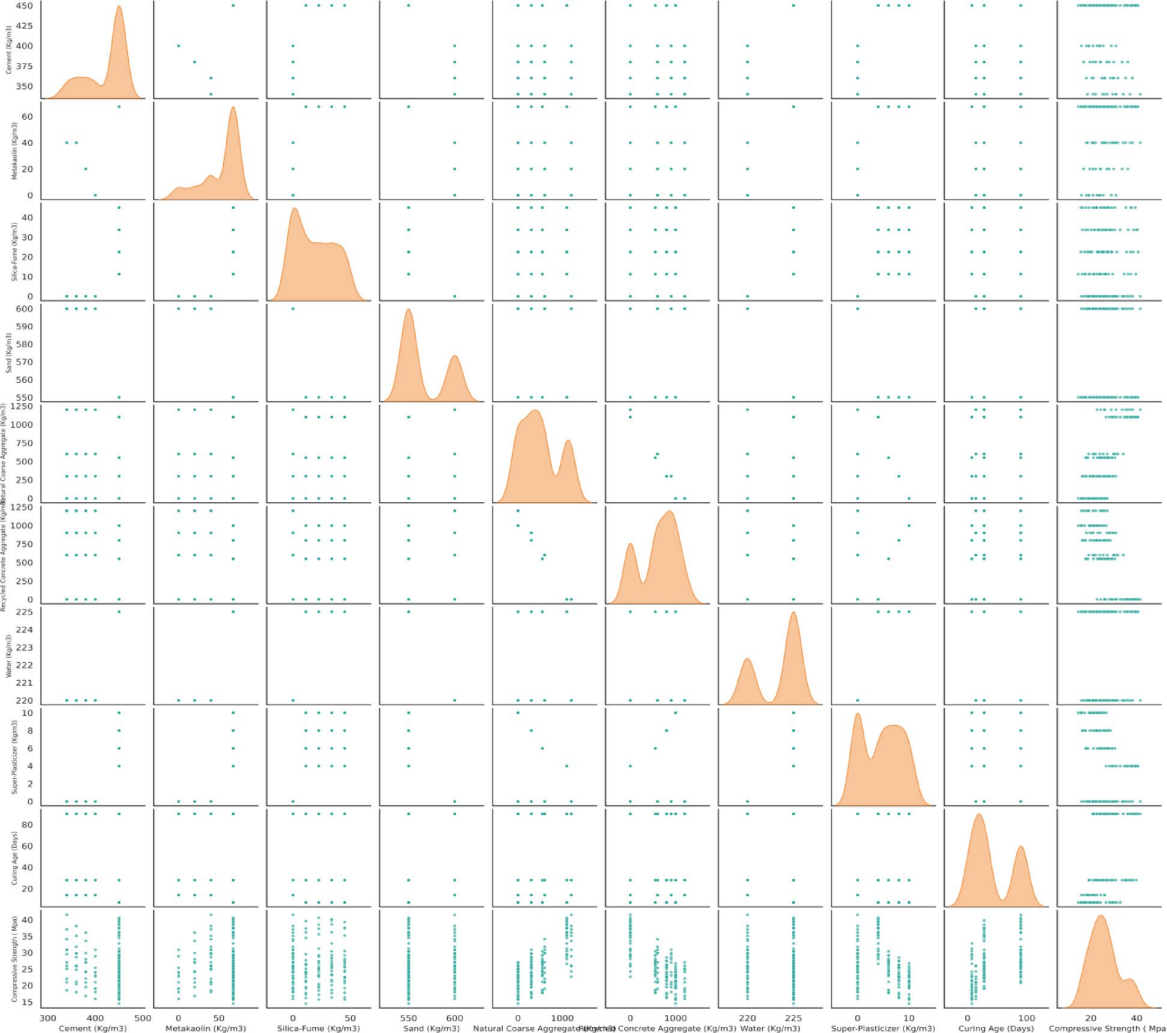
Pairplot of input and output variables.

Figure 6 represents the pair plot matrix, showcasing the relationships between multiple numerical variables related to concrete mix design. Each scatter plot illustrates a bivariate distribution for two specific attributes which offers an insight into possible linear/non-linear relationships. The variable’s diagonal histograms or kernel density plots depict univariate distributions, revealing the individual characteristics of variables such as, but not limited to skewness, kurtosis, and central tendency. The Cement seems to show a skewed distribution which suggests that the dataset contains a greater amount of lower cement values concentrated towards 400 kg/m^3^ cement content. Also, scatter plots on the lower triangle show pairs of variables. A good instance would be the positive correlation between Cement and Compressive Strength (CS); it indicates that the compressive strength of concrete rises with an increase in cement content. This relationship could also simply be computed by the Pearson correlation coefficient which would yield a conclusive value supporting both strength and direction of the assertive evaluation stated above. On the contrary, the scatter plot suggesting a negative correlation between Water and Super-Plasticizer indicates an inverse relationship where increased water content results in reduced super-plasticizer content. Moreover, the variable Water seems to be evenly distributed which implies a less skewed distribution and suggests more consistency of water content across the dataset. A lack of strong observable relationships also appears in some scatter plots, such as Curing Age and certain aggregates suggesting these variables are unlikely to have a significant linear dependency. Outliers can be observed from pair plots in some variables like Curing Age and Super-Plasticizer which tend to affect how correlation and regression analyses are interpreted.

#### Correlation analysis

In order to understand how features are dependent on the target strength factors, requires an evaluation of synergies between variables. Relationship evaluations often assist in developing a predictive model that fits best for the dataset in question. Alongside many other types of metrics, one especially focuses on the Pearson correlation coefficient (r), helping to assess quantitatively if two features and their relationship are associated strongly enough to justify using them together and also flagging if the relationship is positive or negative. This approach can be helpful when analyzing various types of linear relationships^57,58^. The Pearson correlation coefficient (r) is obtained from the covariance (cov) of two considered random magnitude pairs x and y. Covariance is measured as shown in below Eq. 5:

5 $$r = \frac{{cov \left( {x, y} \right)}}{{\sigma_{x} \sigma_{y} }} = \frac{{\mathop \sum \nolimits_{i = 1}^{n} \left( {x_{i} - \overline{x}} \right)\left( {y_{i} - \overline{y}} \right)}}{{\sqrt {\mathop \sum \nolimits_{n = 1}^{n} (x_{i} - \overline{x})^{2} } \sqrt {\mathop \sum \nolimits_{n = 1}^{n} (y_{i} - \overline{y})^{2} } }}$$

where $\overline{x}$ and $\overline{y}$ are the mean of two variables $x$ and $y$ whereas $n$ is the number of a dataset.

**Fig. 7:**
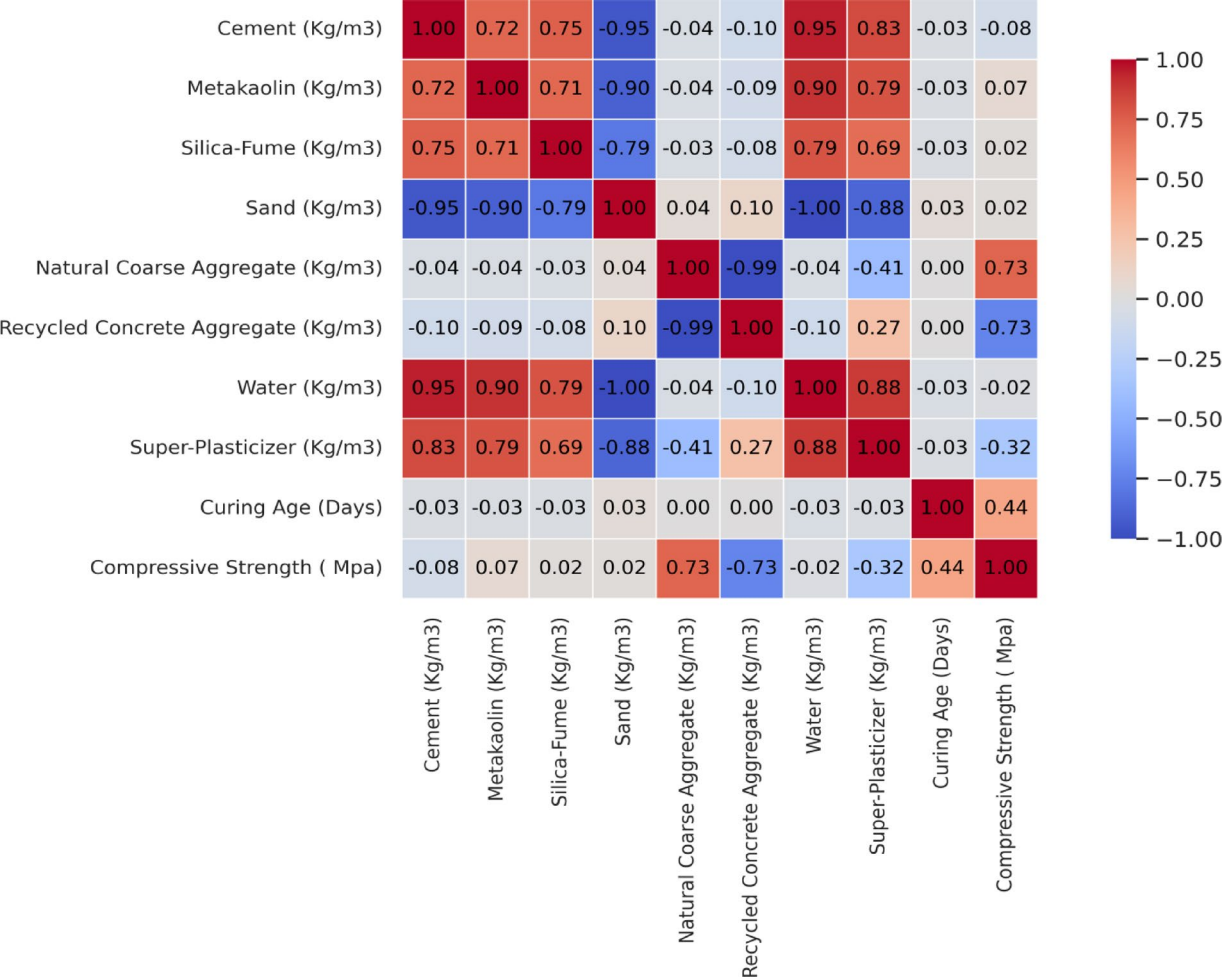
Correlation heatmap of dataset.

With this analysis, valuable insights can be drawn as to whether augments of one variate trigger its partner variable to augment (positive correlation) or regress (negative correlation), or if there is no degree of linear influence at all. Figure 7 presents an analytical heatmap which reveals how each variable correlates with others in the dataset. The heatmap demonstrates that the most notable positive correlations occur among natural coarse aggregates, curing age and compressive strength with correlation coefficients of 0.73 and 0.44 respectively. These values illustrate that the compressive strength (CS) of concrete is primarily determined by the quantity of NCA in the mix, then the curing duration as their support this claim.

Conversely, the strongest negative correlation occurs between recycled concrete aggregates (RCA) and super-plasticizer with values of − 0.73 and − 0.32. This indicates a strong inverse relationship between RCA and super-plasticizer content used in the mix with compressive strength. The other parameters exhibit weak correlations with each other and with compressive strength, suggesting that there are no substantial linear relationships among them. The absence of high collinearity indicates that all eight features capture important and distinct information. Therefore, all these variables are pertinent and can be efficiently utilized for predicting the compressive strength of concrete.

### Machine learning algorithms

**Fig. 8:**
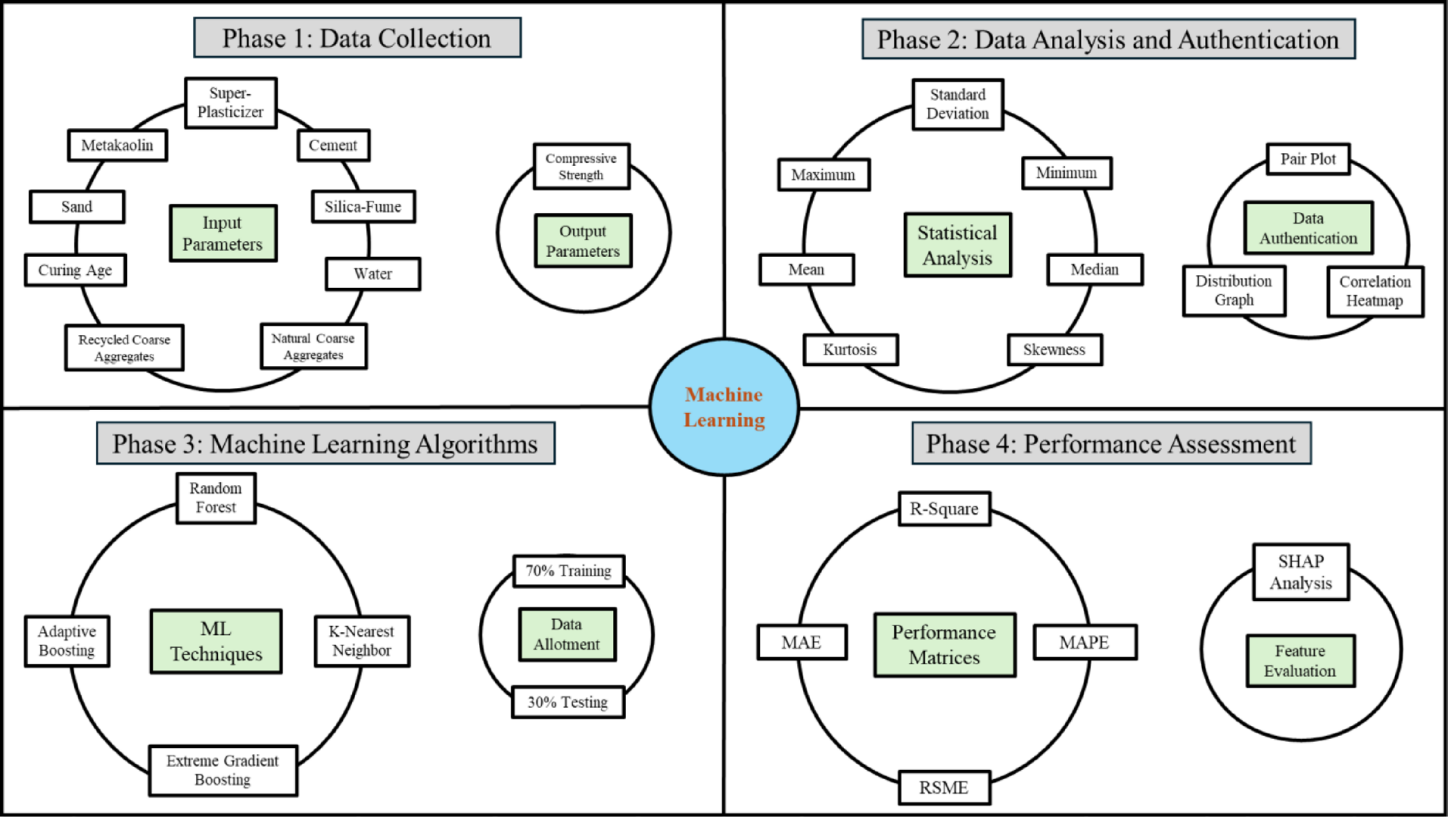
Workflow of machine learning process.

**Fig. 9:**
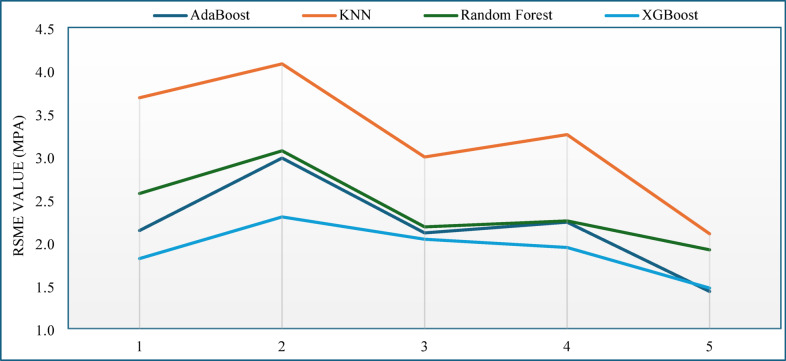
Variation of RSME values for different fold of various machine learning models.

In this research, the compressive strength of concrete was predicted to be using four different machine learning (ML) models which were created and assessed based on varying training-to-testing dataset. The previous researcher relayed for partitioning of the data set on a trial-and-error strategy to find the best ratio that achieves a satisfactory trade-off between accuracy and generalization. At first, the allocation of training versus testing datasets favored training with 90% while testing utilized the remaining 10%. This was further changed in decremented steps of 10%, i.e., increasing proportion for the test set which changed to 80:20, 70:30, and finally 60:40 (as previous studies have suggested^59–61^. A detailed workflow is shown in Fig. 8 below:

The effectiveness of each ML model within these configurations was evaluated using the coefficient of determination (R^2^) for each model’s performance, one of the most accepted metrics for assessing accuracy in regression models^62^. From all schemes dealing with data partitioning, the 70:30 train-test split finally turned out to be the best choice as it achieved the highest R^2^ value as well as minimal difference between training and testing performance. This demonstrates high generalization capability while significantly reducing the risk of overfitting, which is necessary for making reliable predictions on new scenarios^63,64^. Such evidence supports earlier studies that underlined the need to focus on proper data split to achieve a balance between accuracy and evaluation precision in relation to learning and measuring confidence in model performance^65,66^. In this way, an absolute benchmark configuration was set based on a standard 70% training partition size paired with a 30% testing partition size for subsequent model development and assessment. This approach enabled the implementation of ML models designed to predict the compressive strength of concrete with high accuracy across many values of other input variables^67^. However, in this study a fivefold cross-validation (CV) method was used to prevent bias because the model was trained using a single random split of the data as reported by many researchers for best results. The data was split so that 70% was used in training and the remaining 30% was used in testing that was held back as a hold-out set to be used in the final model validation. Under the training subset, K-Fold CV was used to partition the data into five equal parts, with four folds being used in training the model, and one in validation at a time. To get a more accurate estimate of model generalization, the average performance of all folds was then calculated. This method proved to be a great way of minimizing variance in model evaluation in comparison with traditional single-split approaches and to make sure that all the observations of the training set were utilized during training and validation^68,69^. The predictive accuracy of the algorithms was also improved by the integration of the Grey Wolf Optimizer (GWO) to tune the hyperparameters during the cross-validation stage to duly search the best parameter space of each model^70^. The CV analysis outcomes indicated that the performance of the models, particularly the XGBoost, was consistent in different folds, which ensured that the models had a predictive ability that was stable and generalized. The small gap in fold-level the R^2^ and RMSE implied that overfitting was restricted and that it fits unobservable data well. As a result, the CV-GWO approach improved the reliability of the models in addition to offering statistical confidence that observed performance trends were not data-dependent, but sample-dependent. There is observable difference in the values of the RMSE in the different folds which is primarily because of the variation in the data separation and the sensitivity of the different models to the variation as shown in Fig. 9. Regardless of these variations, models XGB and ADB would continually yield the lowest RMSE values, indicating their stability in terms of prediction. Precisely, XGB produced the lowest mean RMSE of 1.912 ± 0.304 MPa then ADB with the second best of 2.18 ± 0.55 MPa, respectively and significantly lower than the RF and KNN with mean RMSE of 2.93 ± 0.44 and 3.22 ± 0.75 respectively. This steady pattern underscores the successful application of boosting algorithms in the identification of complicated nonlinear patterns within the data.

The general statistical result of the models in fivefold cross-validation is given in Table 9. The best predictive performance was realized with XGB (R^2^ = 0.913 + 0.024) followed by ADB (R^2^ = 0.885 + 0.054) and with relatively lower determination coefficients, the results of RF (R^2^ = 0.861 + 0.055) and KNN (R^2^ = 0.747 + 0.116) were recorded. Besides, the values of the normalized mean absolute percentage error (MAPE) were very low, across all the models, indicating that the model predicted all the folds without bias. Generally, XGB and ADB both had high accuracy and reliability with insignificant performance differences, but RF and KNN performed worse with more unstable performance.

**Table 9:** Mean and standard deviation values of evaluation matrices of different ML models.

Evaluation Metrics	XGB	ADB	RF	KNN
R-Square	0.913 ± 0.024	0.885 ± 0.055	0.862 ± 0.055	0.747 ± 0.117
RSME	1.912 ± 0.304	2.180 ± 0.551	2.397 ± 0.441	3.222 ± 0.750
MAE	1.628 ± 0.222	1.843 ± 0.505	2.071 ± 0.378	2.703 ± 0.626
MAPE	6.805 ± 1.140	7.718 ± 2.141	8.581 ± 1.709	11.490 ± 2.950

The best hyperparameters in every machine learning model were identified through the Grey Wolf Optimizer (GWO) and shown in Table 10, to improve the predictive performance of each model. In the case of the XGBoost model, the optimal model was obtained with a learning rate value of 0.0206, a maximum depth of 2, and 672 estimators (subsample and colsample by tree value of 0.509 and 0.500 respectively), and regularization parameters (alpha = 0.253 and lambda = 0.958) minimized overfitting. The optimal results of AdaBoost were on 709 estimators, and the learning rate was 0.0207, which showed a high model generalization. The 120 estimators, maximum depth of 9 and small leaf and split sizes (2 and 4 respectively) in the Random Forest model generated consistent and correct predictions. In KNN, k = 2, uniform weighting and Manhattan distance (*p* = 1) gave the best results, indicating that it is sensitive to the structure of local neighborhoods. These tuned parameters were then evaluated and compared on models on cross-validation.

**Table 10:** Tunned hyperparameter of different ML models after fivefold cross validation.

Tunned Hyperparameters	XGB	ADB	RF	KNN
Number of Neighbor	–	–	–	2
Distance Metrices (p)	–	–	–	1
Maximum Tree Depth	2	–	4	–
Learning Rate	0.0206	0.7030	–	–
Number of Estimator (Tree)	672	166	106	–
Minimum Samples per Split	–	–	2	–
Minimum Samples per Leaf (Child Samples)	–	–	1	–
Maximum Features (col sample by tree)	0.5	–	–	12
Row Sampling Ratio (Sub sample)	0.5094	–	–	–
Regularization L1 (α)	0.2531	–	–	–
Regularization L2 (λ)	0.9581	–	–	–
Random State	42	42	42	–

#### Random forest

**Fig. 10:**
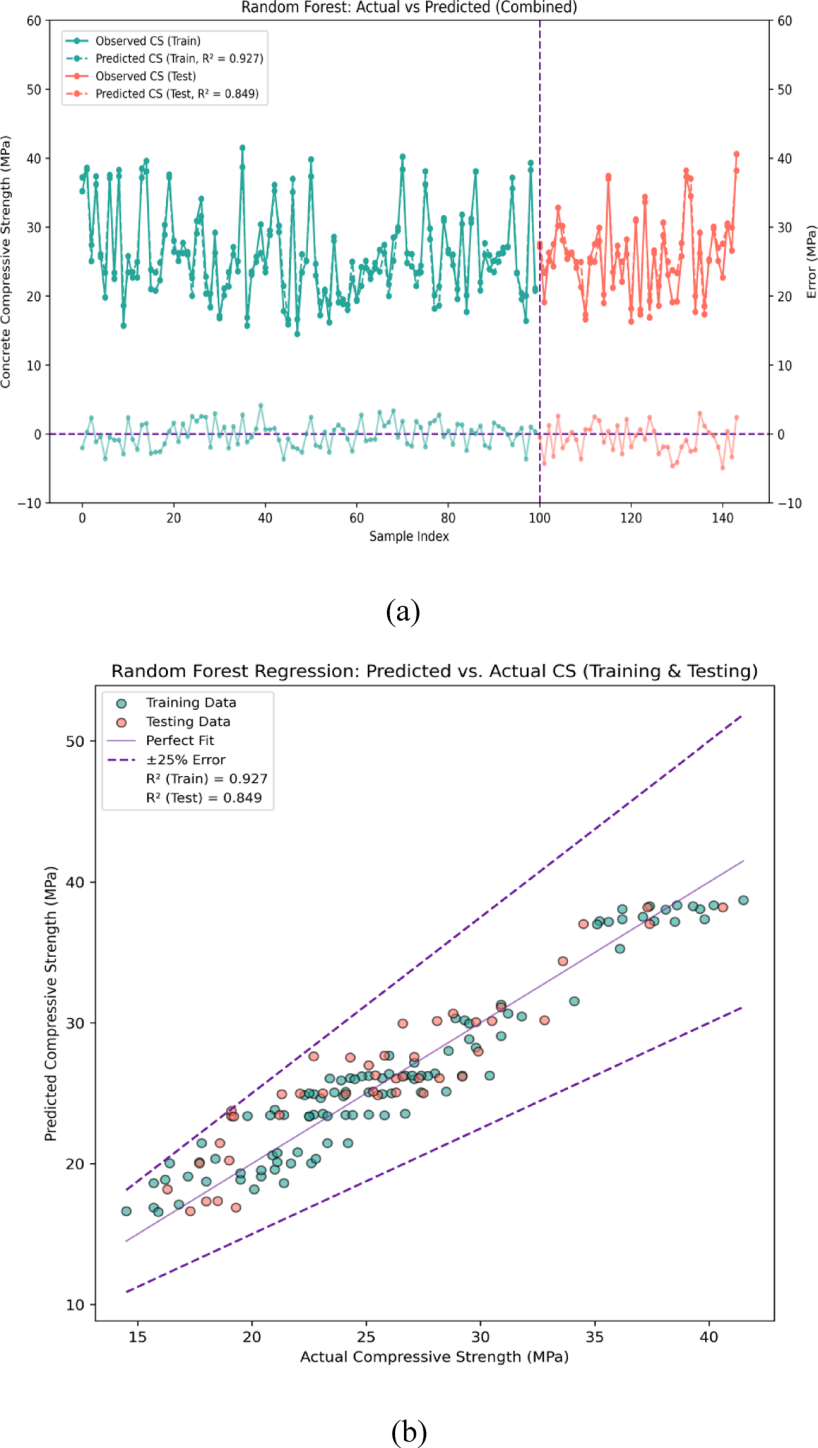
(**a**) Performance evaluation for prediction of CS using RF (error graph). (**b**) performance evaluation for prediction of CS using RF (regression graph).

Figure 10a and b showcase the application of the Random Forest (RF) regression model on predicting the compressive strength (CS) of Metakaolin-Silica fume based Recycled aggregates concrete. In Fig. 10a, we can see a composite depiction of actual versus predicted values of compressive strength for both training and testing datasets. The observed and fitted values for the training set are colored green while those of the testing set are in red. The vertical dashed separation indicates where the training data ends and testing data begins. The secondary y-axis demonstrates the prediction error for each sample, which is the model’s performance validation. The RF model shows strong accuracy in fitting during training (R^2^ = 0.927) and reasonable accuracy in generalization through testing (R^2^ = 0.899). For the training data, the maximum error is 3.49 MPa and for the testing data, the corresponding values is 4.14 MPa.

Figure 10b shows the scatter plot illustrates the relationship between predicted compressive strength values and actual strength values for both training and testing datasets. The data points are clustered closely along the 1:1 reference line (perfect fit) which is shown by the solid purple line; the dashed lines indicate a ± 25% error margin. Most of the data points sit within this threshold. The robustness of the model is also indicated by the R^2^ values: 0.949 for training and 0.899 for testing. 100% of the testing data points are within the ± 25% error margin, showcasing the RF model’s strong accuracy and alignment with experimental values.

#### K-nearest neighbor

**Fig. 11:**
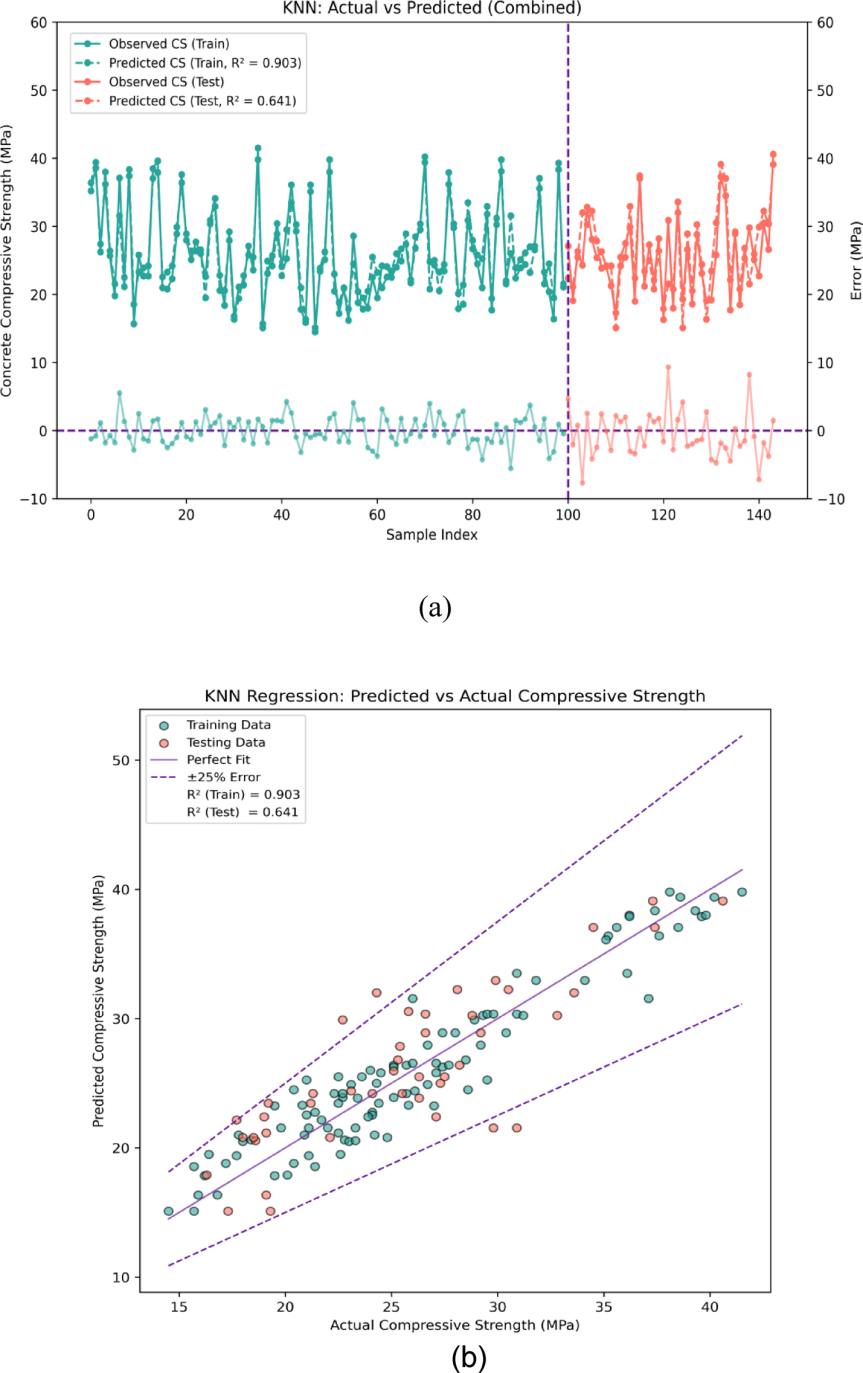
(**a**) Performance Evaluation for Prediction of CS using KNN (Error Graph). (**b**): Performance Evaluation for Prediction of CS using KNN (Regression Graph).

Using the KNN regression model, the compressive strength (CS) prediction and evaluation of recycled concrete aggregate (RCA) is done in Fig. 11a and b. As shown in Fig. 11a, there is both positive and negative error in prediction of RCA compressive strength based on the actual values. In terms of error metrics, the KNN model for the training dataset shows a minimum error of 0.08 MPa, a maximum error of 8.92 MPa, and an average error of 3.72 MPa. In comparison, the KNN model testing dataset showed a minimum of 0.03 MPa, a maximum error of 9.65 MPa, and an average of 5.74 MPa. Overall, these findings indicate that the KNN model serves as a good estimate of compressive strength but the model is more reliable during training than in testing. Remarkably, in the training stage, around 70% of the errors are below 1 MPa, suggesting the model accurately predicts compressive strength for a large portion of the dataset. Roughly 20% of the errors are within 1–5 MPa, showing some level of predictive accuracy. The final 10% of the predictions have much greater error, exceeding 5 MPa, indicating outliers or more complex relationships that the model has difficulty capturing.

The error distribution is analyzed further in the error scatter plot of Fig. 11b, where ± 25% error boundaries are set for both training and testing phases. This area demonstrates the extent to which prediction accuracy exists, which is quite good since most data points in both training and testing phases fall within this area. More than 60 percent of the predicted data points clustered around the linear fit (1:1) line indicating the model is accurate. This is, however, accompanied by some data points diverging from the 1:1 line, suggesting some model underfitting especially in the testing phase where error is larger than the ± 25% threshold. Analyzing model performance using the coefficient of determination, R^2^, is useful. In the case of the training dataset, it leads us to an R^2^ value of 0.903, meaning 83.8% of the variance in the compressive strength can be accounted for by the KNN model. However, for the out-of-sample R^2^ in the testing phase this drops to 0.641 suggesting greater variability in predictions and hinting that the model is less generalizable to new data.

#### Extreme gradient boosting

**Fig. 12:**
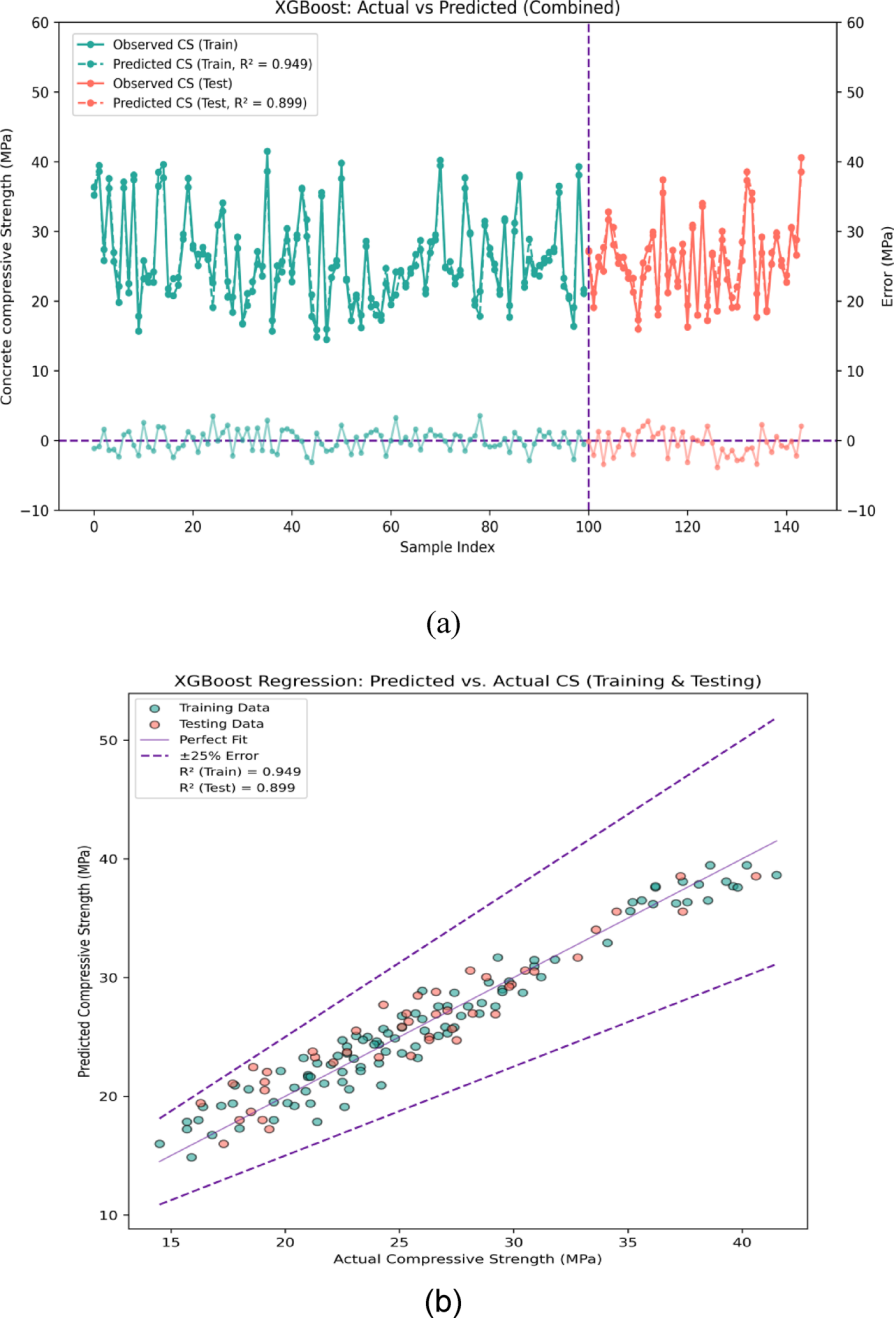
(**a**) Performance evaluation for prediction of CS using KNN (error graph). (**b**) Performance evaluation for prediction of CS using XGB (regression graph).

The outcomes and expected results of the compressive strength with the application of XGBoost model algorithm are shown in Fig. 12a. The XGBoost model forecasts has the following errors: 0.06 MPa, 3.64 MPa, and 4.21 MPa for minimum, maximum, and mean errors respectively during training stage. The minimum error of 0.06 MPa shows the model’s close working with predicted and actual values while 3.64 MPa maximum error indicates the model’s large occasional deviations which may be due to outliers or complicated data points. The average error of 4.21 MPa gives a significant summary about the accuracy of the model which tells us that the model can predict the compressive strength values with moderate accuracy. In the same vein, XGBoost model’s lowest, highest and mean error values during testing are 0.04 MPa, 7.12 MPa, and 5.14 MPa respectively which indicates the model is reasonably consistent in its performance on new, previously unseen data but does seem to have a higher mean error. About 50% of the errors are below 1 MPa which suggests accuracy for quite a number of the predictions made. Almost 30% of errors between the intervals of 1 to 3 MPa indicates some ability to accurately estimate CS values for a large portion of the dataset. Roughly 20% of the errors greater than 3 MPa indicates a stronger deviation from the predicted values for more complicated associations among the input variables and compressive strength due to non-linear or complex structures.

Figure 12b shows the comparison of the expected data to the actual data sets. This scatter plot shows that more than 95% of the predicted data points for the XGBoost model are in close agreement with the linear fit 1:1 line which indicates strong correlation and accuracy in prediction. In addition, the XGBoost model seems to outperform the KNN and RF models as these other models have a greater number of predicted data points that are beyond the ideal linear fit. The few outliers that exist, with the model predicted values beyond the error band of ± 25 MPa, imply that the model is likely struggling due to some underlying complexities within the data. For the XGBoost model, the R^2^ values are 0.949 for training and 0.899 for testing. The R^2^ value of 0.949 for the training stage suggests that the model captures approximately 94.9 percent of the variation in the observed compressive strength values, which is exemplary performance. The R^2^ ratio in the testing phase reflects that the model still accurately predicts 87.1% of the testing data although some reduction in performance is observed, likely due to overfitting on the training set or inherent variability in the testing data.

#### Adaptive boosting

**Fig. 13:**
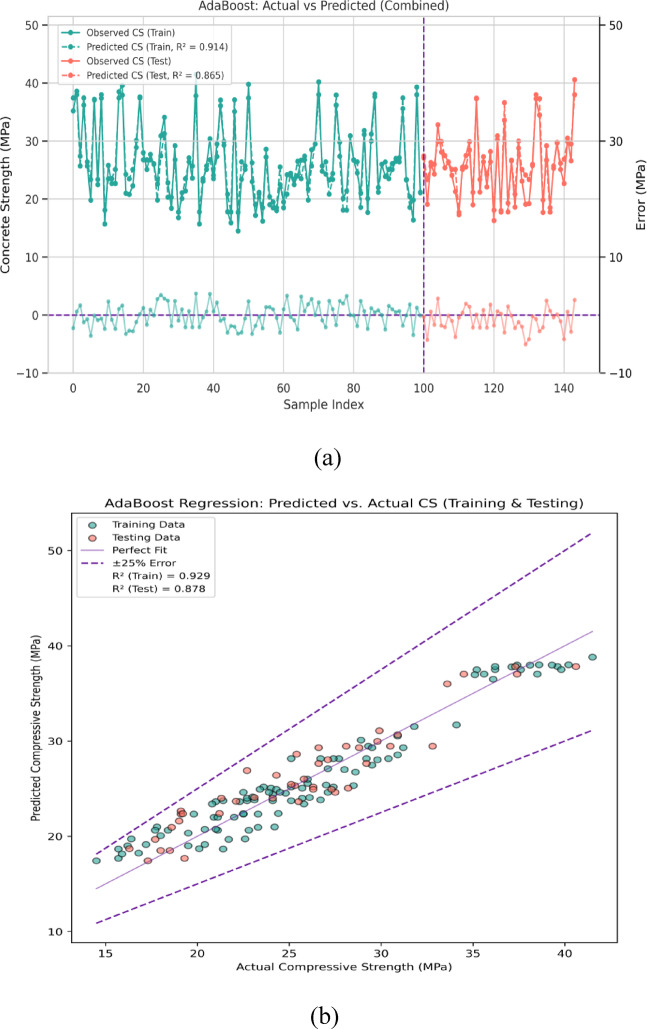
(**a**) Performance Evaluation for Prediction of CS using ADB (Error Graph). (**b**) Performance Evaluation for Prediction of CS using ADB (Regression Graph).

In Fig. 13a, the actual versus predicted outcomes for the compressive strength (CS) of concrete using the AdaBoost algorithm are displayed and shows how the actual and predicted CS values are distributed, along with errors in prediction for both the training and testing datasets. For the training stage, the AdaBoost model has a minimum, maximum, and mean prediction error of 0.03 MPa, 4.35 MPa, and 2.60 MPa, respectively. Likewise, for the testing phase, the model has minimum, maximum, and mean errors of 0.02 MPa, 4.59 MPa, and 2.20 MPa, respectively. These findings reinforce the strong predictive accuracy achieved by the AdaBoost algorithm, capturing the variability of CS for previously unseen data informed by the training dataset. More specifically, around 35% of errors are predicted to be less than 1 MPa, while nearly 50% of errors fall within the 1–3 MPa range, and 15% exceed 3 MPa. This particular distribution of errors suggests that as a whole, the AdaBoost model performs well in predicting CS, but in niche cases, the severe magnitude error overshadows slight overperformance.

As shown by the scatter plot, the observed and predicted CS values align closely along a 1:1 line, demonstrating the adequacy of the AdaBoost model in comparison to the actual CS values. Figure 13b visually demonstrates that the error margins for both training and testing datasets are bound within a 25% range, encapsulating over 99.9% of the predicted data points. This clearly illustrates the AdaBoost model’s remarkable linear compliance. The R^2^ value from the AdaBoost model indicates a metric of 0.929 for the training dataset which means it explains 92.9% of the variance in compressive strength (CS) values. For the testing dataset, the R^2^ drops slightly to 0.878 which means 87.8% of the variance is explained. Although there is a slight decrease in R^2^ from training to testing, the model still demonstrates strong predictive performance. With high R^2^ values, it can be confirmed that AdaBoost effectively models CS and generalizes well to unseen data. Comparing AdaBoost with models like KNN and XGBM emphasizes AdaBoost’s strong performance and reliability in modeling concrete compressive strength. These findings underscore AdaBoost’s efficiency for practical applications, predicting with precision and dependability.

#### Model’s performance comparison

**Fig. 14:**
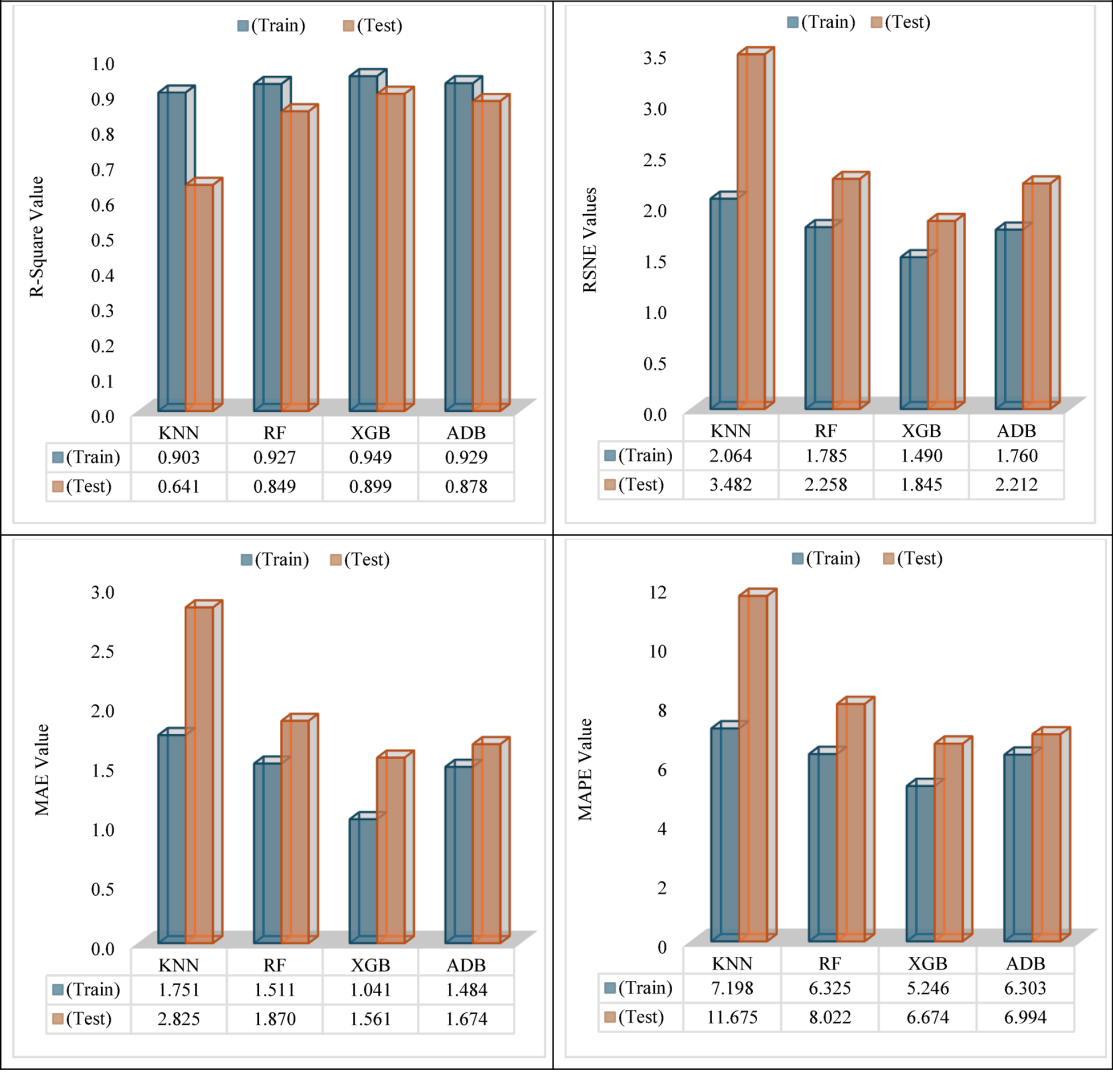
Evaluation metrices of machine learning algorithms for various models.

Figure 14 shows the performance evaluation of machine learning (ML) models with the statistical metrics including R-Square, RMSE, and MAE. The R-Square values demonstrating XGB’s capability relative to other models serving as metrics for pattern extraction in data exhibit R-Square values of 0.949 on the training set and 0.899 on the test set. It can be interpreted from these values that the XGB model creates a good fit for the data learned as well as performs adequately on the unseen data. This ability to generalize is further endorsed by the RMSE where XGB demonstrates a low loss of 1.490 on training and 1.845 on testing. For many regression tasks, RMSE serves as a benchmark to judge the accuracy of predicted values, the smaller the number provided, the more accurate the predictive capabilities are. In comparison, Adaptive Boosting demonstrates a slightly lower RMSE on the training set with 1.760 and its RMSE on the test set is 2.212 which indicates overfitting on the training data making XGB a more stable choice. While RF achieves a remarkable result with a MAE value of 1.511 for training data and 1.870 for testing data, it still underperforms in comparison to ADB and XGB in other aspects of evaluation. XGB does capture the lowest MAEs giving it the lowest absolute errors of 1.041 for training but 1.561 for testing which is still lower than ABD with a value of 1.674, thus XGB have the lowest MAEs for testing. Also, it is observed that RF to have greater RMSE values than ADB in the testing data where it reaches 1.511. In addition, XGB does well in explaining variance (R-Square) with a training score of 0.949 and testing score of 0.899 and still overrun ADB in MAE and MAPE metrics. For ADB, the test MAE is 1.484, and MAPE is 6.303 which is higher than XGB’s 1.041 and 5.246. MAPE provides a ratio-based comparison of prediction accuracy that favors lower values. Even with XGB having better MAPE numbers, ADB’s considerable performance compensates for it, however XGB remains inferior to ADB when it comes to predictive accuracy and model robustness averaged across all the metrics.

KNN, in this case, performs the worst in all four metrics. Its R-Square values of 0.903 for training and 0.641 for testing suggest that KNN explains the least variance in data, especially in the test set which performs much worse. KNN’s RMSE is the worst out of all models with a test set value of 2.064 for training and 3.482 for testing. This shows a lack of predictive accuracy. Its MAE and MAPE are the highest as well, supporting that KNN is not capturing the data patterns as compared to other algorithms. In short, Extreme Gradient Boosting (XGB) performs better than all other models as it computes visibly the highest R-Square values, the lowest RMSE, and strong results for MAE and MAPE. This makes XGB the most dependable and efficient model for the data set used dataset. Although ADB and RF show strengths in some of the metrics, XGB stands out in predictive power, accuracy, and generalization and KNN is the worst of the four with high error metrics coupled with low R-Square values make this model the worst option.

#### SHAP analysis

**Fig. 15:**
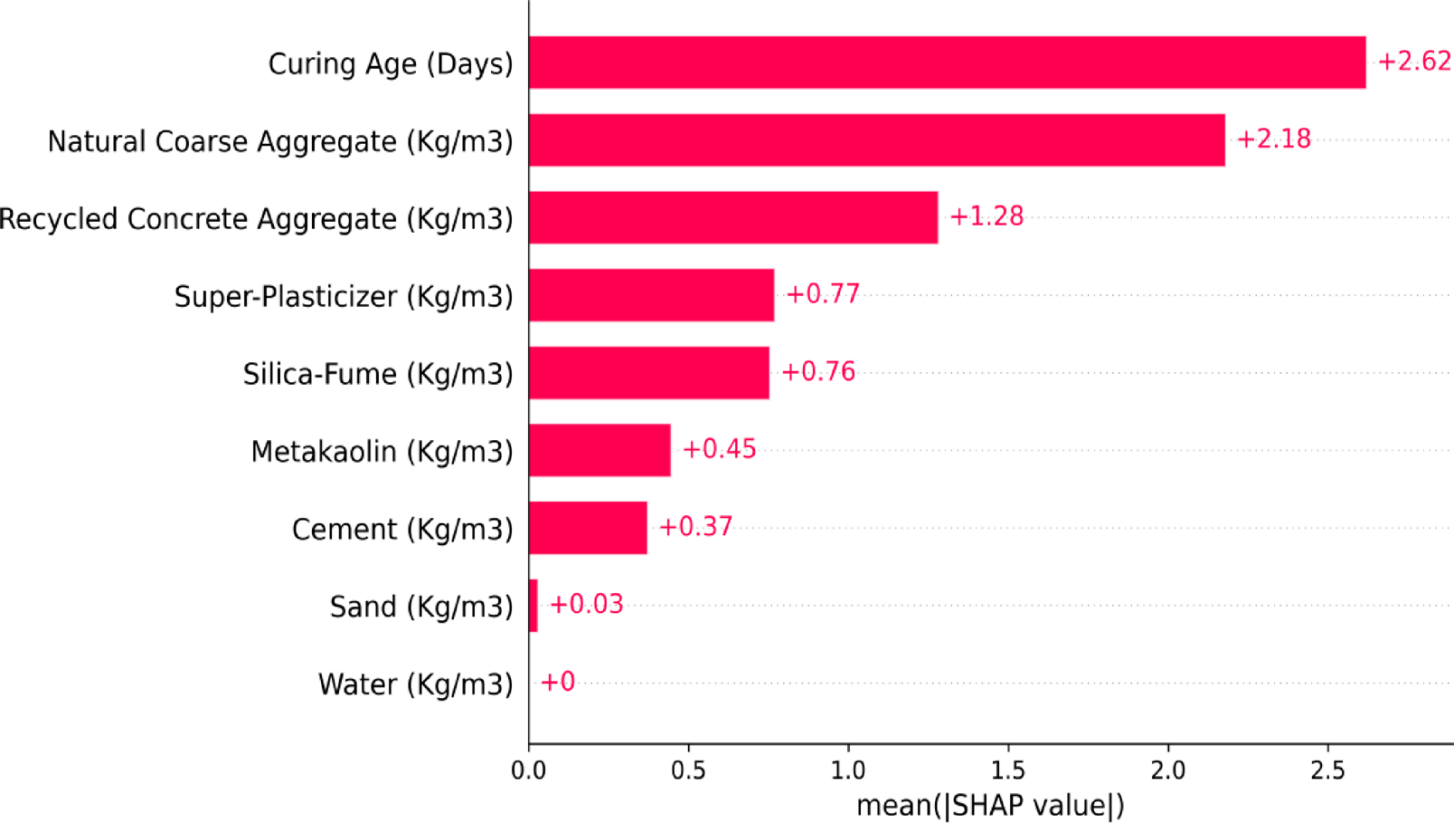
SHAP bar plot.

Shapley Additive Explanations (SHAP) analysis offers a comprehensive approach to understanding the contributions of various features in machine learning (ML) models, helping to explain each feature’s role in predictions. Figure 15 displays a SHAP bar plot showing the average absolute value of Shapley metrics and how they contribute to the prediction of the compressive strength (CS) of Metakaolin-Silica Fume based Recycled Aggregate Concrete. It becomes evident from the plot that Curing Age (CA) and Natural Coarse Aggregate (NCA) are two of the most relevant features with CA offering a maximum gain of + 2.62 and NCA offering a lesser but still significant + 2.18 to the prediction. These results reveal that curing age and the NCA of the concrete significantly determine compressive strength. Other input features such as RCA and SP do contribute positively but are relative to a smaller degree. In this case, RCA and SP have values of + 0.128 and + 0.77 which display the beneficial impact on compressive strength. Meanwhile Silica fume (SF) Metakaolin (MK) and Cement also have lower but positive contributions with SHAP values of + 0.76, + 0.45, and + 0.37 respectively. Sand exhibit much lesser (+ 0.03) while Water exhibit no direct effect on the model’s output, with a SHAP value of 0, indicating no relationship with compressive strength prediction. Moreover, RCA impacts on output begin less than anticipated, although its influence is still positive.

**Fig. 16:**
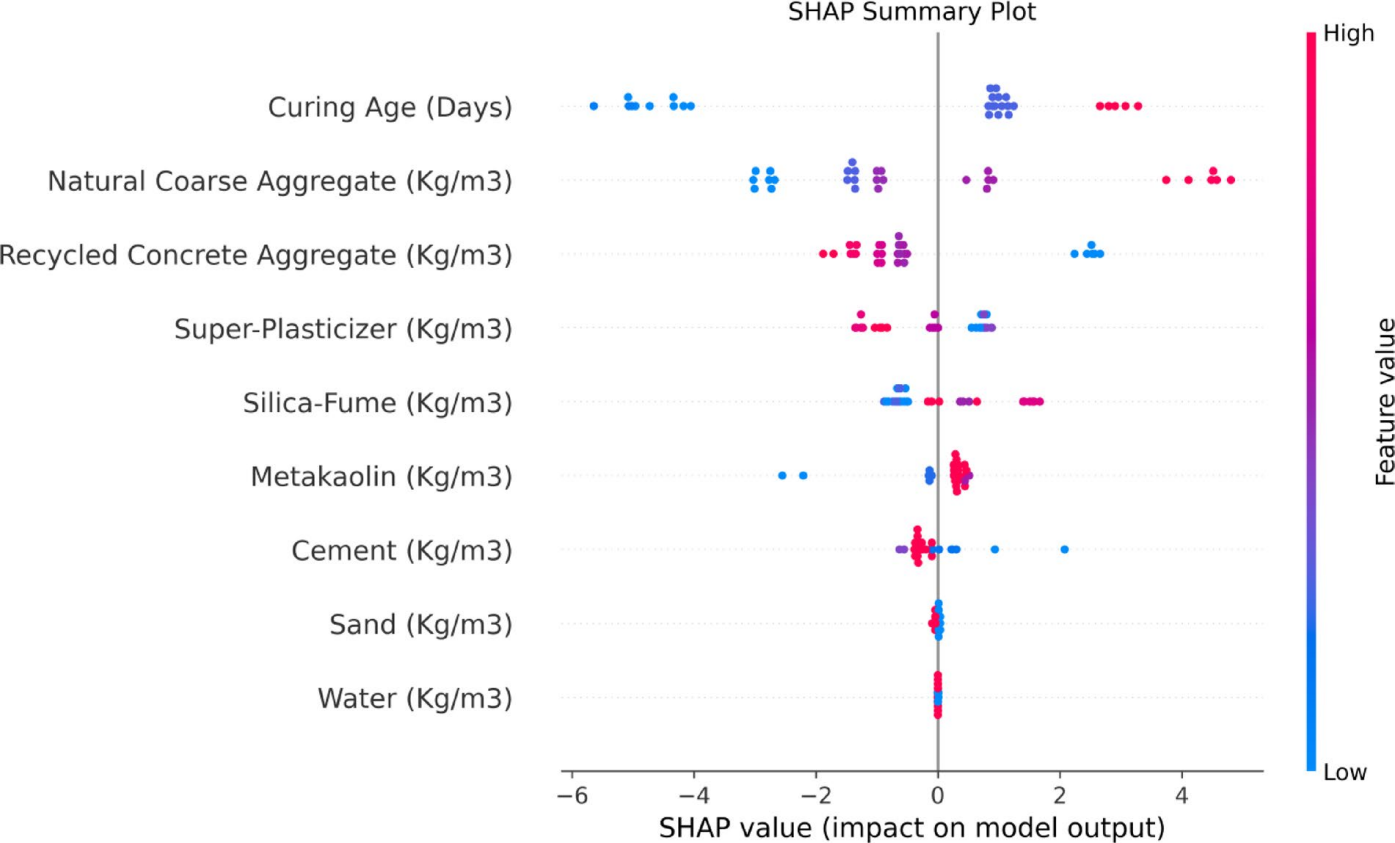
SHAP summary plot.

To examine the relationship between these variables and their compressive strength influence, SHAP summary plots are provided in Fig. 16. This plot helps illustrate the effect of each feature in relation to model output where rank order is preserved in the vertical axis. The horizontal axis communicates the range of SHAP values of each feature’s effect with respect to the output. Each sample is represented by a dot colored according to the feature value which is low (blue) to high (red). Notably, Curing Age and Natural Coarse Aggregate positively and strongly influence compressive strength as evidenced by the clustering of red (high values) at the upper range of SHAP values. As noted, the increase in Curing Age and NCA shows a positive relationship with compressive strength, reiterating the role of aging and the amount of NCA used in developing concrete’s compressive strength. The hegemony of the curing age is very much correlated to the experimental findings, in which compressive strength was continuously increasing until 28 days and proved that evolution of the strength was mainly dominated by hydration and pozzolanic reactions. Equally, the SF content SHAP value was positively correlated, where the higher the SF to 7.5–10 percent, the higher the CS became due to better interfacial transition zone (ITZ) and lower micro voids, which is in line with the experimental trends. On the other hand, the adverse SHAP effect of increased RCA content is also in favor of the experimentally determined decrease in strength caused by weaker adhered mortar and increased water absorption of recycled aggregates. Moreover, the intermediate effect of moderate levels of MK and water-to-binder ratio in SHAP analysis is additionally supported by the results of experimental work, in which MK-modified samples of RCA mixes showed a higher strength recovery than the samples without any modification. Such overlap between SHAP understanding and an experimental study confirms the trustworthiness of the hybrid methodology and that the machine learning model assumed the real-world processes behind the strength difference in untreated and treated RAC.

## Conclusions

The construction industry has begun to realize the value of Artificial Intelligence in forecasting the mechanical characteristics of concrete, especially compressive strength. This paper has compared the performance and accuracy of various machine learning (ML) models with varied material inputs using the experimental and past literature. Four regression algorithms namely AdaBoost, K-Nearest Neighbors, Random Forest and XGBoost were explored and their hyperparameters was tuned using K-Fold CV and optimized with GWO and the subsequent main conclusions were made:

- The compressive strength of concrete is affected in a way that more the RCA has been replaced, the lesser the compressive strength. The negative impact is however reduced by the application of SCMs and R100-SF7.5 with 15% metakaolin treatment gives satisfactory strength (25 MPa), which is the minimum pavement construction requirement. This improvement in the experimental results further validated by the ANOVA which that all the *P*-Values are too less than 0.05.
- The inclusion of untreated and treated RCA with SF and MK contributed greatly to abrasion resistance. The optimum SF content (7.5%) was found to improve surface durability, which surpassed the natural aggregates and offered sustainable and durable pavements of RCA.
- The results of the correlation analysis revealed that the age at which the curing occurred and NCA were strongly positively related to compressive strength, whereas superplasticizer and RCA were negatively correlated.
- The highest predictive accuracy was obtained with XGBoost model, which has R^2^ values of 0.949 (training) and 0.899 (testing) which was higher than in other models. Whereas AdaBoost and RF provided satisfactory results but could not compete with XGBoost and ADB regarding the generalization of results in the test set.
- The values of RMSE, MAPE, and MAE indicated that all models have a high predictive generalization because of low error rates with XGBoost being the most reliably predictive model across all datasets.
- SHAP analysis showed that curing age and NCA had the highest scores of importance (+ 2.62 and + 2.18), which means that they significantly influence the prediction, and cement and sand had a negligible impact on compressive strength.

The paper explores ML tools in the prediction of the compressive strength (CS) of RAC. However, still some areas needs to be explored further like combining the material properties and concrete chemistry may enhance the accuracy of the model. The increase in datasets and optimization of hybrid and deep learning models should be extended to the future because other factors such as temperature, corrosion, and chemical resistance were not analyzed comprehensively. The suggested ML framework is also applicable in evaluating other RCA properties like strength, durability, porosity, shrinkage, cost, and carbon emissions.

## Data Availability

The data is available from the corresponding author upon request.
